# A multilevel analysis identifies the different relationships between amino acids and the competence of oocytes matured individually or in groups

**DOI:** 10.1038/s41598-020-73225-7

**Published:** 2020-09-30

**Authors:** Rasoul Kowsar, Alireza Mansouri, Nima Sadeghi, Mohammad Heidaran Ali Abadi, Seyed Mehdi Ghoreishi, Khaled Sadeghi, Akio Miyamoto

**Affiliations:** 1 Department of Animal Sciences, College of Agriculture, Isfahan University of Technology, 84156-83111 Isfahan, Iran; 2 Global Agromedicine Research Center (GAMRC), Obihiro University of Agriculture and Veterinary Medicine, Obihiro, Hokkaido 080-8555 Japan; 3 FKA, Animal Husbandry and Agriculture Co, Isfahan, Iran; 4 Department of Animal Sciences, College of Agriculture, Shiraz University, Shiraz, Iran

**Keywords:** Biomarkers, Predictive markers, Metabolomics, Computational models

## Abstract

High-protein diets contribute to an increase in urea follicular concentrations associated with decreased fertility. Urea has been shown to interfere with the epidermal growth factor (EGF)/EGFR system, which has been shown to have a beneficial effect during in vitro maturation (IVM) of oocytes. Of note, the number of cumulus-oocyte complexes (COCs) in the maturation medium can change the maturation and the developmental competence of COCs. Therefore, it was hypothesized that, the presence of urea and EGF may have a differential effect on the depletion/appearance of AAs and competence of COCs matured individually (I-IVM system) or in groups (G-IVM system). In the G-IVM system, COCs increased consumption (depletion) of AAs compared with other groups in the presence of high-level urea (40 mg/dl) + EGF (10 ng/ml). In the I-IVM system, the non-cleaved COCs depleted more AAs than the cleaved COCs, in particular in the presence of urea. The combination of urea and EGF increased the depletion of AAs in the G-IVM system. However, the EGF abrogated the urea-induced depletion of AAs by the I-IVM COCs. The use of *N*-acetyl-l-cysteine as an EGFR inhibitor canceled urea-induced depletion of AAs. This shows the inhibiting effect of urea over the EGF/EGFR system. In the presence of urea + EGF, COCs had a lower degree of developmental competence than control in both I- and G-IVM systems. Arginine had the best predictive power to identify highly competent COCs in the G-IVM system, while glutamine was the best predictor of the cleavage in the I-IVM system. In conclusion, this multi-level study shows that COCs matured individually or in groups may have different association with AAs metabolism. These findings provide new insights into the relationships between AA metabolism and the subsequent developmental competence of COCs.

## Introduction

Follicular fluid, which is in close proximity to maturing oocyte, contains the epidermal growth factor (EGF)^1,2^. Previous studies have shown that bovine oocytes express the EGF receptor (EGFR) at all stages of maturation^3^. In addition, cumulus cells (CCs) have been shown to mediate the positive effects of EGF on bovine oocyte maturation and subsequent embryonic development after in vitro fertilization (IVF)^2–4^. Continuous activity of EGFR is essential for the chronic phosphorylation of the ERK1/2 downstream signaling molecules that have been shown to be necessary for oocyte maturation and cumulus expansion^5^. EGFR signaling activates MAPK, decreases the cGMP rate of the granulosa cells and promotes the closure of the gap junction of the follicle-enclosed cumulus-oocyte complexes (COCs) through phosphorylation of the connexins^6,7^. This, in turn, disrupts the flow of meiotic inhibitors, i.e., cAMP and cGMP, into oocytes and removes meiotic arresting signals^6–8^.

Interestingly, it has been shown that urea and its derivatives inhibit the function of EGFR tyrosine kinase^9–11^. It can therefore be concluded that urea would alter the metabolic effects of EGF on COCs, i.e., the metabolism of amino acids (AAs), and decrease the subsequent developmental competence of oocytes. Studies reported similar concentrations of urea in the blood and follicular fluids of dairy cows^12^. It may be caused by a passive transfer of urea from blood to body fluids, i.e., follicular fluids^13^. High protein diets (17–19% crude protein) are significantly associated with an increased concentration of urea in the blood and reproductive fluids of healthy dairy cows^14^. High protein diet is typically used to stimulate and promote high milk production in early lactation of dairy cows^14^. Blood urea nitrogen (BUN) levels greater than 19 mg/dl are directly associated with decreased reproductive efficiency in healthy dairy cows^14^. A growing body of evidence supports the detrimental effect of high levels of urea on the competence of bovine oocytes, in vivo and in vitro^15–17^. Moreover, urea increases the consumption of AAs by CCs, denuded oocytes (DOs) and COCs^17^. Hemmings et al. reported a concomitant increase in AA consumption and a decrease in the developmental competence of bovine and human oocytes^18,19^.

The function of system L amino acid transporters has been reported to be reduced in oocytes from the germinal vesicle stage to metaphase II^20^. This means that the functions of AA transporters can be changed as the oocyte grows. The state of maturation and patterns of AA transporters can therefore change the biochemical results of experimental treatments. Therefore, the synchronization or separation of oocytes from a particular stage (i.e., MII stage) should be considered in order to minimize the stage-specific effects of oocytes on AA depletion/appearance. Oocytes synchronization is mediated by components that accumulate and activate the cAMP signaling pathway in oocytes^21^. However, the activity of certain AA transporters is decreased from the M1 to the MII stage in which the oocytes are moved to a quiet stage^22^. In addition, the accumulation of cAMP within oocytes can alter the function of AA transporters^23^. In this study, depletion/appearance of AAs was reported in 24 h-spent maturation media to circumvent the potential effects of in vitro synchronization or oocyte development on AA metabolism. Moreover, COCs were specifically recovered from 7–8 mm follicles for oocytes with the same maturation stage as possible. Lonergan et al*.* reported that the oocytes collected from follicles of similar size had a close maturation stage^24^. Importantly, oocytes cultured individually have been reported to have a lower maturity and developmental competence than oocytes matured in groups. It has been shown that CCs and oocytes maintain the supply of paracrine and autocrine factors for each other, especially in cultures with more than 20 COCs, which will lead to oocyte growth^25^.

Therefore, we first hypothesized that the depletion/appearance of AAs and the developmental competence of COCs would change in the presence of urea and EGF in the maturation medium. It was also presumed that the individual or group maturation of COCs may alter their reactions to urea and EGF. In this study, a multi-step approach, including univariate, bivariate, multivariate and network analysis, was used to identify similarities and associations between AAs and oocyte competence.

## Results

### Intracellular urea content in bovine denuded oocytes (DOs)

**Figure 1:**
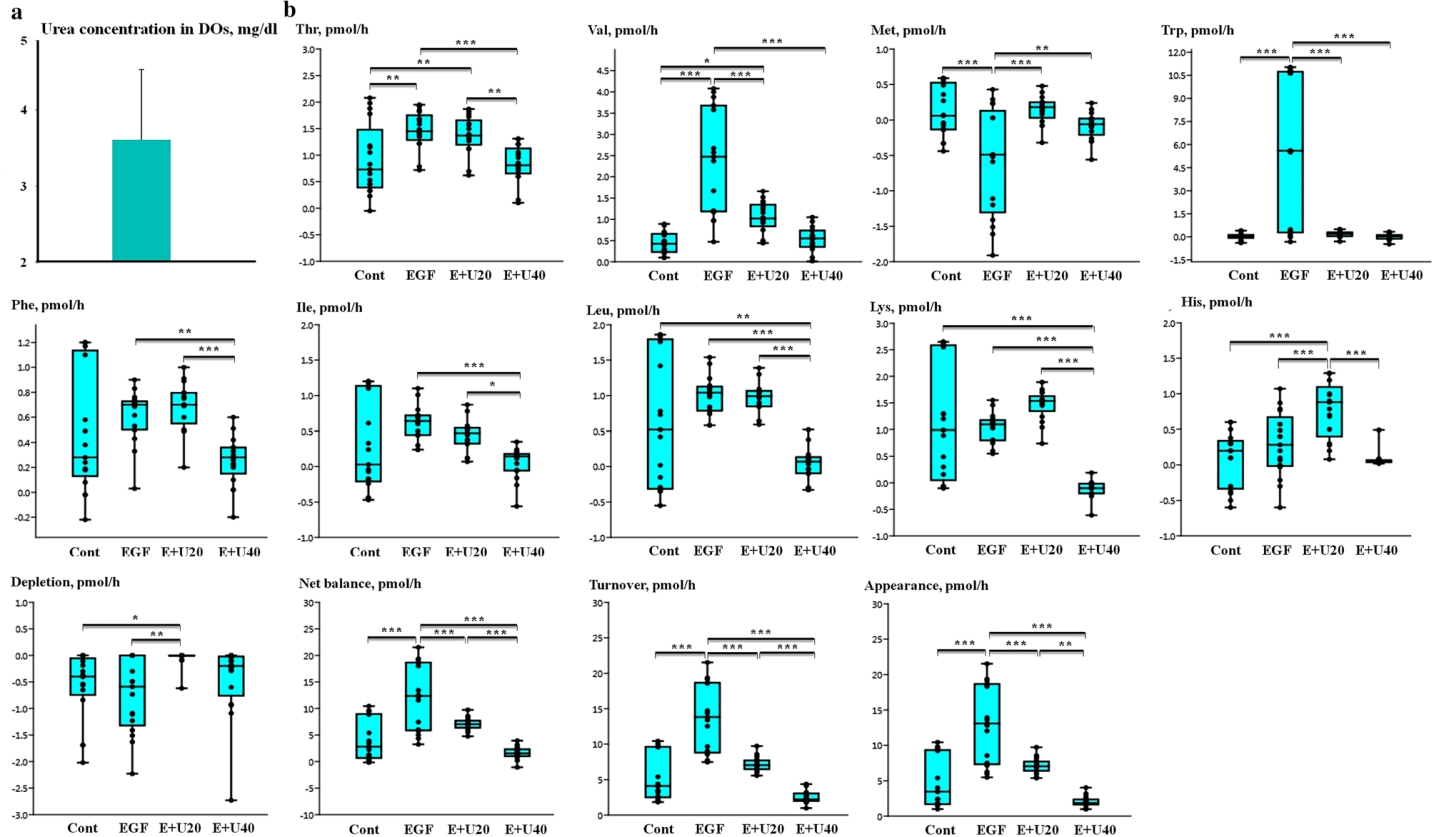
**(a) **4-h incubation of denuded oocyte (DOs) with 40 mg/dl of urea. This experiment was conducted to determine the intracellular urea content of the DOs (3 replications, 25 DOs per each replication, mean ± SD). **(b) **Using the group-IVM culture (10 COCs per droplet), the 24-h incubation of COCs with urea and EGF altered the depletion/appearance of essential amino acids (EAAs). Differences between the concentration of AA in the test medium (after 24 h of incubation) and the fresh medium were used to calculate the depletion or appearance of each AA. Negative and positive values indicate the “depletion” and “appearance” of the EAAs, respectively. The “total depletion”, “total appearance”, “total net balance” and “total turnover” of EAAs was calculated using data from the depletion/appearance of the EAAs grouped into six replicates (based on the 6 time points of IVM conducted, Supplementary Fig. 1, Phase 1). The negative (depletion) or positive (appearance) values obtained for EAAs were summed up for each time point. Boxes range from the 25th to the 75th percentile of the distribution of values for each group. The median is represented by the line inside the box. The whiskers represent the maximum and minimum data point inside 1.5 times the interquartile range. Dots denote values outside the range of adjacent values. The data was obtained from 17 measurements and the medians were analyzed using the Kruskal–Wallis test followed by the Dunn’s multiple comparison test. Urea and EGF are shown as U and E, respectively. Cont: control group; EGF group; 10 ng/ml EGF; E + U20: 10 ng/ml EGF + 20 mg/dl urea; and E + U40 group: 10 ng/ml EGF + 40 mg/dl urea group; Asterisks indicate significant differences between groups: *: *p* < 0.05; **: *p* < 0.01; ***: *p* < 0.001.

The results revealed a concentration of 3.43 ± 1.4 mg/dl (mean ± SD) of urea in DOs cultured for 4 h with 40 mg/dl of urea (Fig. 1a).

### Calculation of depletion or appearance of amino acids (AAs)

Differences between the concentration of AA in the test medium (after 24 h of incubation) and that of the fresh medium were used to calculate the depletion or appearance of each AA^18,19^. Negative values indicate that AAs have disappeared from the maturation medium after 24-h incubation (depletion). Positive values indicate that AAs appeared at higher concentrations in the maturation medium after 24-h incubation (appearance). For the calculation of “total depletion”, “total appearance” of AAs, the negative (depletion) or positive (appearance) values obtained for AAs were summed up^18,19^ (Supplementary Fig. 1, Phase 1). Next, the “total net balance” of all AAs was calculated by subtracting “total depletion” from “total appearance”. In addition, the “total turnover” of all AAs was determined by the sum of “total depletion” and “total appearance”. Therefore, there were 6 data (6 time points) for the analysis of “total depletion”, “total appearance”, “total net balance” and “total turnover” of AAs.

### Univariate analysis to evaluate the impact of EGF and urea on the depletion/appearance of EAAs by COCs matured in groups

Compared to COCs matured in the presence of a high urea level (40 mg/dl) plus EGF (10 ng/ml), higher amounts of Thr (*p* < 0.001), Phe (*p* < 0.01), Ile (*p* < 0.001), Val (*p* < 0.001), Leu (*p* < 0.001), Lys (*p* < 0.001), and Trp (*p* < 0.001) were released by COCs in the presence of 10 ng/ml EGF (Fig. 1b). Furthermore, Ile and Lys were significantly depleted by COCs matured in the presence of EGF and a high level of urea; however, these EAAs were produced by COCs in the presence of EGF (*p* < 0.001). COCs matured in the presence of EGF (10 ng/ml) consumed more Met than other experimental treatments (*p* < 0.01). It was also noted that the total appearance (*p* < 0.001), total turnover (*p* < 0.001) and total net balance (*p* < 0.001) of EAAs was higher in EGF-incubated COCs, but the presence of EGF plus a high level of urea during in vitro maturation (IVM) removed this effect from the EGF (Fig. 1b).

### Univariate analysis to determine the effect of EGF and urea on the depletion/appearance of non-EAAs, semi-EAAs and all AAs by COCs matured in groups

**Figure 2:**
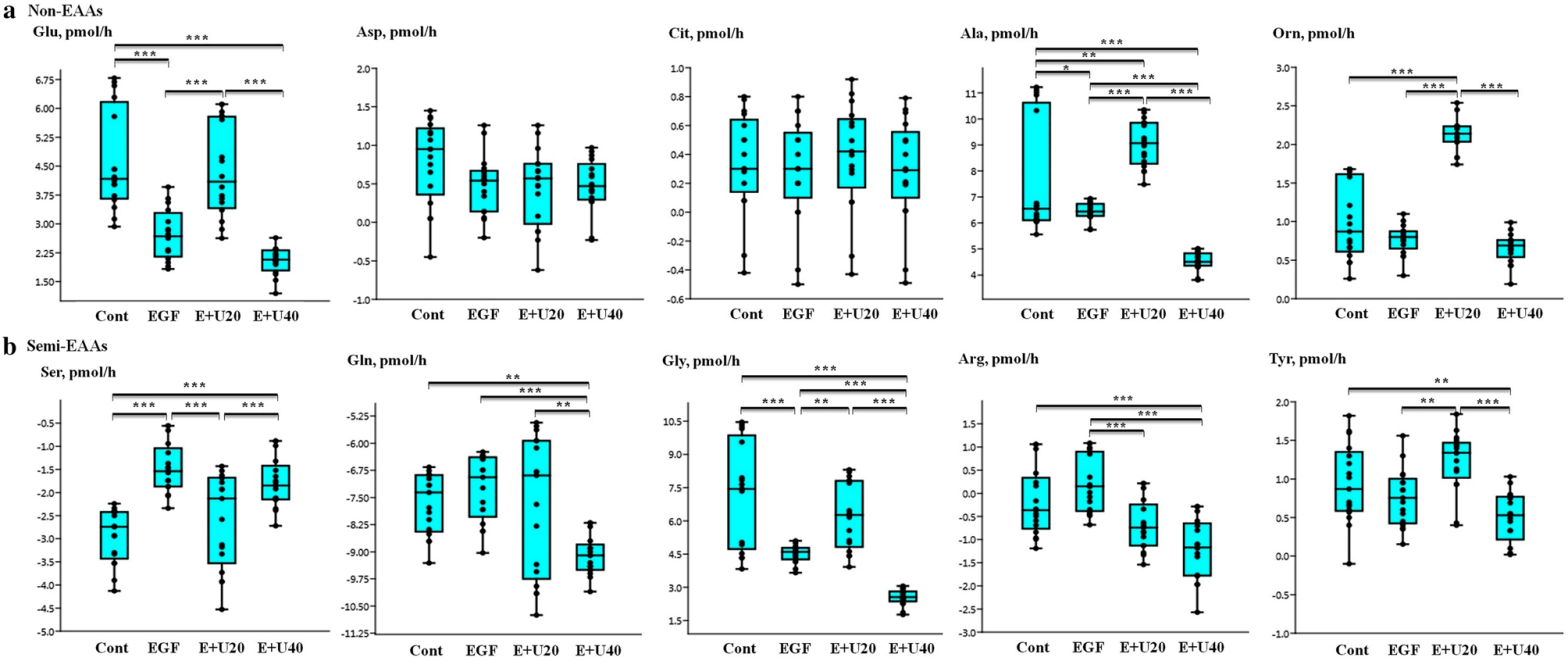
Using the group-IVM culture (10 COCs per droplet), the 24-h incubation of COCs with urea and EGF altered the depletion/appearance of** (a) **non-essential amino acids (non-EAAs) and** (b) **semi-EAAs. Differences between the concentration of AA in the test medium (after 24 h of incubation) and the fresh medium were used to calculate the depletion or appearance of each AA. Negative and positive values indicate the “depletion” and “appearance” of the AAs, respectively. Boxes range from the 25th to the 75th percentile of the distribution of values for each group. The median is represented by the line inside the box. The whiskers represent the maximum and minimum data point inside 1.5 times the interquartile range. Dots denote observations outside the range of adjacent values. The data was obtained from 17 measurements and the medians were analyzed using the Kruskal–Wallis test followed by the Dunn’s multiple comparison test. Urea and EGF are shown as U and E, respectively. Cont: control group; EGF group: 10 ng/ml EGF; E + U20 group: 10 ng/ml EGF + 20 mg/dl urea; and E + U40 group: 10 ng/ml EGF + 40 mg/dl urea. Asterisks indicate significant differences between groups: *: *p* < 0.05; **: *p* < 0.01; ***: *p* < 0.001.

**Figure 3:**
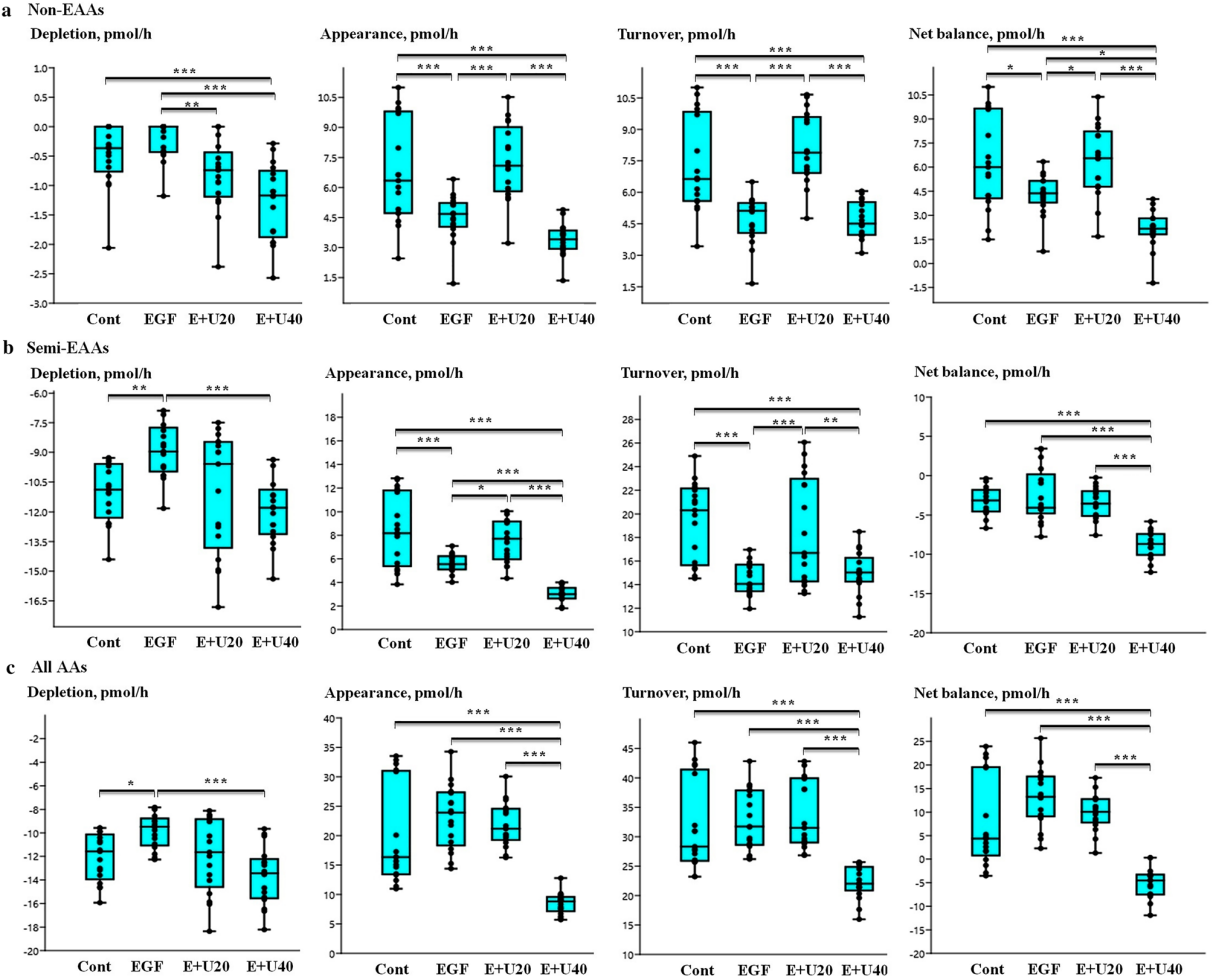
Total depletion, total appearance, total turnover and total net balance of **(a) **non- essential amino acids (EAAs), **(b) **semi-EAAs, and** (c) **all AAs of bovine COCs. COCs were matured for 24 h in the group-IVM culture supplemented by EGF and urea. The “total depletion”, “total appearance”, “total net balance” and “total turnover” of EAAs was calculated using data from the depletion/appearance of the EAAs grouped into six replicates (based on the 6 time points of IVM conducted, Supplementary Fig. 1, Phase 1). Then, the negative (depletion) or positive (appearance) values obtained for EAAs were summed up for each time point. Therefore, there were 6 data from 6 time points for the analysis of “total depletion”, “total appearance”, “total net balance” and “total turnover” of AAs. The data was obtained from 17 measurements and the medians were analyzed using the Kruskal–Wallis test followed by the Dunn’s multiple comparison test. Boxes range from the 25th to the 75th percentile of the distribution of values for each group. The median is represented by the line inside the box. The whiskers represent the maximum and minimum data point inside 1.5 times the interquartile range. Dots denote observations outside the range of adjacent values. Urea and EGF are shown as U and E, respectively. Cont: control group; EGF group: 10 ng/ml EGF; E + U20: 10 ng/ml EGF + 20 mg/dl urea; and E + U40 group: 10 ng/ml EGF + 40 mg/dl urea group. Asterisks indicate significant differences between groups: *: *p* < 0.05; **: *p* < 0.01; ***: *p* < 0.001.

Compared with COCs matured during IVM at a high urea level (40 mg/dl) plus EGF (10 ng/ml), COCs matured in the presence of EGF (10 ng/ml) produced more Ala (*p* < 0.001) and Gly (*p* < 0.001) (Fig. 2a,b). In comparison, COCs matured in the presence of EGF plus a high level of urea increased (*p* < 0.001) the consumption of Arg and Gln (Fig. 2b). In addition, COCs matured in a culture medium with a high level of urea plus EGF had a higher net balance of non-EAAs (*p* < 0.001) than the EGF-incubated COCs (Fig. 3a). COCs matured in the presence of a high urea level plus EGF had a higher total depletion (*p* < 0.001) and the lower total appearance (*p* < 0.001) of semi-EAAs and all AAs compared to the EGF group (Fig. 3b,c). COCs incubated with a high urea level plus EGF showed a significant decrease (*p* < 0.001) in the total net balance of semi-EAAs/all AAs and in the total turnover of all AAs (Fig. 3b,c).

### Developmental competence of COCs and mRNA expression of genes in day-7 blastocysts resulting from bovine COCs matured in groups

**Figure 4:**
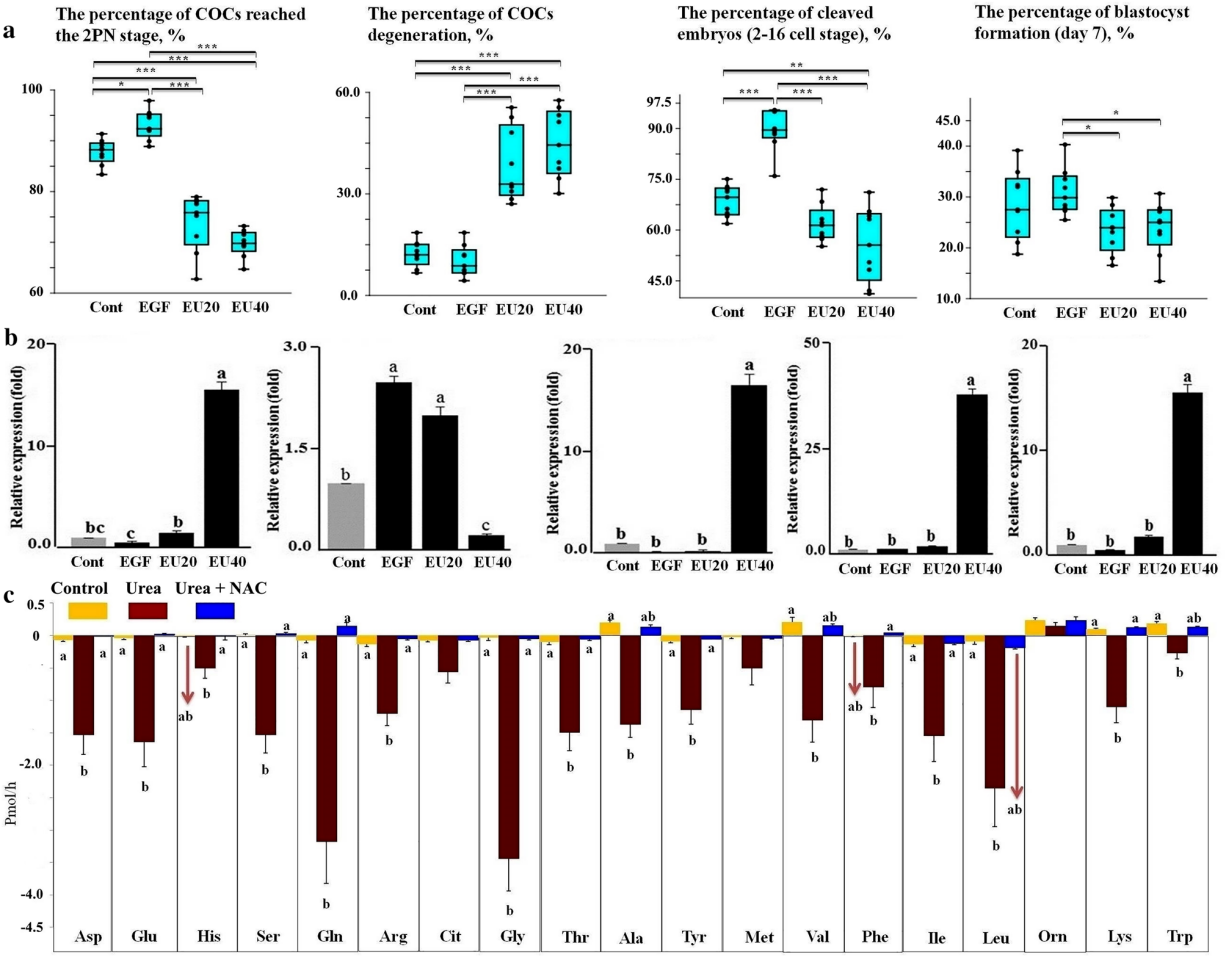
**(a) **Developmental competence of COCs matured for 24 h in a group-IVM culture supplemented with urea and EGF. Boxes range from the 25th to the 75th percentile of the distribution of values for each group. The median is represented by the line inside the box. The whiskers represent the maximum and minimum data point inside 1.5 times the interquartile range. Dots denote observations outside the range of adjacent values. The data was obtained from 17 measurements and the medians were analyzed using the Kruskal–Wallis test followed by the Dunn’s multiple comparison test. Asterisks indicate significant differences between groups: *: *p* < 0.05; **: *p* < 0.01; ***: *p* < 0.001. All parameters relating to developmental competence of the COCs were shown in relation to the initial number of COCs cultured in the maturation medium. 2-PN: two-pronuclear zygote; Cleavage: 2–16 cell stage; Degeneration: oocyte degeneration by day 3 post-insemination; Blastocyst: day-7 blastocyst. **(b) **Relative mRNA expression of *BAX*, *BCL2*, *NANOG*, *OCT4*, and *DNMT1* in day-7 blastocysts. Blastocysts (7 to 8 blastocysts per treatment) derived from COCs matured for 24 h for 24 h in a group-IVM culture supplemented by urea and EGF. Data are means ± S.E.M and are representative of nine replicates analyzed using the one-way ANOVA followed by Tukey's multiple comparison test (Supplementary Fig. 1, Phase 3)**.** **(c)** 24-h incubation of COCs with urea and N-acetyl-l-cysteine (an EGFR inhibitor) using a group-IVM culture. It was done to test whether urea affects depletion/appearance of amino acids through EGFR. Data are means ± S.E.M and are representative of 6 replicates. Different letters indicate significant differences (*p* < 0.05). One-way ANOVA followed by Tukey's multiple comparison test was used.

All parameters of developmental competence were reported on the basis of the initial number of COCs cultured in the maturation medium. The results showed a significant increase in 2-PN *(p* < 0.05) and the percentage of COCs reached the cleavage stage (2–16 cell stage, *p* < 0.001) by applying EGF to the maturation medium (Fig. 4a). Adding EGF and urea to the culture medium during IVM decreased the percentage of 2PN, cleavage, and blastocyst formation (*p* < 0.01), whereas the percentage of oocyte degeneration (by day 3 post-insemination) increased (*p* < 0.001) compared to the EGF group (Fig. 4a).

The mRNA expression of *BCL2* was up-regulated in day-7 blastocysts resulting from COCs matured in the presence of EGF compared to the control group (*p* < 0.05, Fig. 4b). However, the presence of high urea level and EGF during IVM resulted in *BCL2* mRNA down-regulation in the resulting blastocysts (*p* < 0.001). In the presence of EGF and a high level of urea in the medium during IVM, the mRNA expressions of *BAX*, *NANOG*, *OCT4*, and *DNMT1* were significantly up-regulated (*p* < 0.001) in the resulting blastocysts (Fig. 4b).

### Linear and quadratic impact of urea on the depletion/appearance of AAs by COCs and subsequent developmental competence

Linear and quadratic analyses were conducted as a result of linear use of urea in this study (0, 20, and 40 mg/dl, Supplementary Table 1). With an increase in urea, there was a quadratic decrease in the appearance of Glu (*p* = 0.02), His (*p* < 0.01), Gly (*p* = 0.04), Cit (*p* = 0.02), Ala (*p* < 0.01), Tyr (*p* = 0.03), Lys (*p* < 0.05) and Orn (*p* < 0.0001) (Supplementary Table 1).

Total appearance, total net balance and total turnover of non-EAAs (*p* = 0.009) as well as total turnover of semi-EAAs (*p* < 0.05) decreased quadratically with an increase in urea levels. The appearance of Val (*p* = 0.006) and Trp (*p* = 0.03) and the total appearance (*p* < 0.001), total net balance (*p* = 0.004) and total turnover (*p* = 0.0006) of EAAs as well as 2-PN (*p* < 0.0001), cleavage (*p* < 0.0001) and the percentage of blastocyst formation (as a proportion of the initial cultured COCs) decreased linearly (*p* < 0.0001) with increasing urea levels. Total depletion of non-EAAs (*p* = 0.009) and the percentage of COCs degeneration by day 3 post-insemination increased linearly (*p* < 0.0001) with an increase in urea levels (Supplementary Table 1). Total appearance, total net balance and total turnover of all AAs and total appearance of semi-EAAs decreased linearly (*p* < 0.001, *p* < 0.0001, *p* < 0.05, and *p* < 0.01, respectively) and quadratically (*p* = 0.006, *p* = 0.008, *p* < 0.05, and *p* = 0.0009), with increasing urea levels. Arg depletion increased linearly (*p* < 0.01) and quadratically (*p* = 0.005) with an increase in urea levels (Supplementary Table 1).

### Depletion/appearance of AAs by COCs matured individually (within each treatment)

As seen in the control group (Table 1), the depletion of Trp (*p* < 0.01), Ile (*p* < 0.05), Lys (*p* < 0.05), Cit (*p* < 0.05), Asp (*p* < 0.05), Arg (*p* < 0.05), Gly (*p* < 0.05), Tyr (*p* < 0.05), and Gln (*p* < 0.05) was higher in the non-cleaved COCs than in the cleaved COCs. In addition, the total depletion and turnover of all AAs as compared to cleaved COCs was increased by non-cleaved COCs (*p* < 0.05).

**Table 1:** Depletion/appearance of amino acids by COCs matured the individual-IVM culture.

	Control, n = 90		EGF, n = 87		Urea, n = 93		Urea + EGF, n = 96	
	Cleaved, 63/90 (70%)^**#**^	Non-cleaved, 27/90 (30%)	Cleaved, 58/87 (66.7%)	Non-cleaved, 29/87 (33.3%)	Cleaved, 37/93 (39.8%)	Non-cleaved, 56/93 (60.2%)	Cleaved, 43/96 (44.8%)	Non-cleaved, 53/96 (55.2%)
**Essential AAs (EAAs)**								
Thr	− 0.06 ± 0.02^AB^	− 0.14 ± 0.05^a^	− 0.35 ± 0.09^A^	− 0.50 ± 0.01^bc^	− 1.14 ± 0.04^C^	− 1.84 ± 0.31^b^	− 0.21 ± 0.05^AB^	− 0.54 ± 0.09^ab^
Val	0.16 ± 0.02^A^	0.26 ± 0.08^a^	− 0.51 ± 0.18^AB^	− 0.5 ± 0.09^ab^	− 0.84 ± 0.27^B^	− 1.75 ± 0.19^b^	− 0.46 ± 0.02^AB^	− 0.68 ± 0.04^ab^
Met	− 0.01 ± 0.00	− 0.05 ± 0.01	− 0.18 ± 0.01	0.12 ± 0.03*	− 0.28 ± 0.21	− 0.72 ± 0.20	0.04 ± 0.01	− 0.17 ± 0.02*
Trp	0.11 ± 0.01^AB^	0.25 ± 0.01^a^**	0.0 ± 0.0^AB^	− 0.81 ± 0.05^a^*	− 0.17 ± 0.06^B^	− 0.37 ± 0.09^b^	0.35 ± 0.05^AB^	0.19 ± 0.03^ab^
Ile	− 0.09 ± 0.01	− 0.19 ± 0.03^a^*	− 0.64 ± 0.05^AB^	− 0.33 ± 0.03	− 0.95 ± 0.36	− 2.14 ± 0.05^b^*	− 0.17 ± 0.06	− 0.72 ± 0.01^ab^*
Leu	− 0.05 ± 0.02^A^	− 0.14 ± 0.05^a^	− 0.95 ± 0.20^AB^	− 1.33 ± 0.11^b^	− 1.54 ± 0.43^B^	− 3.15 ± 0.40^c^	− 0.93 ± 0.09^AB^	− 1.34 ± 0.07^bc^
His	0.0 ± 0.02	− 0.02 ± 0.01^a^	− 0.04 ± 0.01^A^	0.06 ± 0.02	− 0.33 ± 0.17	− 0.67 ± 0.04^b^	0.08 ± 0.02	− 0.04 ± 0.02^a^
Phe	− 0.03 ± 0.01	0.01 ± 0.01^a^	− 0.27 ± 0.11^AB^	0.17 ± 0.02	− 0.50 ± 0.24	− 1.08 ± 0.31^b^	− 0.08 ± 0.03	− 0.29 ± 0.06^ab^
Lys	0.05 ± 0.01^AB^	0.14 ± 0.01^ab^*	0.24 ± 0.01^A^	− 0.63 ± 0.07^b^**	− 0.57 ± 0.02^AB^	− 1.62 ± 0.10^b^*	0.23 ± 0.04^A^	− 0.03 ± 0.01^ab^*
Dep	− 0.24 ± 0.02^A^	− 0.55 ± 0.11^a^	− 2.94 ± 0.03^A^	− 3.28 ± 0.01^b^	− 6.63 ± 1.87^B^	− 13.34 ± 1.91^b^*	− 1.86 ± 0.29^AB^	− 3.78 ± 0.38^a^*
App	0.34 ± 0.01	0.69 ± 0.15^a^	0.25 ± 0.01^AB^	1.17 ± 0.08*	0.30 ± 0.21	0.0 ± 0.0^b^	0.69 ± 0.14	− 0.23 ± 0.03^ab^*
Net	0.10 ± 0.03^A^	0.14 ± 0.05^b^	− 2.69 ± 0.03^BC^	− 2.10 ± 0.06^ab^	− 6.32 ± 2.05^B^	− 13.34 ± 1.91^a^*	− 1.17 ± 0.14^AB^	− 3.55 ± 0.41^c^*
Turn	0.57 ± 0.02^B^	1.25 ± 0.05^b^*	3.19 ± 0.03^B^	4.49 ± 0.10^b^	6.93 ± 0.1.7^A^	13.34 ± 1.91^a^*	2.56 ± 0.44^AB^	4.01 ± 0.35^ab^
**Non-EAAs**								
Cit	− 0.04 ± 0.01	− 0.11 ± 0.02^a^*	− 0.17 ± 0.03^A^	− 0.22 ± 0.02	− 0.28 ± 0.16	− 0.83 ± 0.11^b^*	− 0.50 ± 0.08	− 0.46 ± 0.02^ab^
Ala	0.18 ± 0.03^BC^	0.22 ± 0.04^ab^	0.51 ± 0.18^AB^	6.69 ± 0.18^a^**	− 1.29 ± 0.06^C^	− 1.44 ± 0.24^b^	2.59 ± 0.22^AB^	1.27 ± 0.17^a^*
Glu	− 0.02 ± 0.0^B^	− 0.05 ± 0.02^a^	− 0.44 ± 0.12^AB^	3.05 ± 0.16^a^**	− 0.92 ± 0.11^B^	− 2.35 ± 0.31^b^*	0.24 ± 0.04^B^	− 0.09 ± 0.03^a^*
Asp	− 0.03 ± 0.01^A^	− 0.12 ± 0.01^a^*	− 0.25 ± 0.05^A^	− 0.28 ± 0.06^ab^	− 1.05 ± 0.07^B^	− 2.01 ± 0.31^b^*	− 0.04 ± 0.01^A^	− 0.33 ± 0.07^ab^*
Orn	0.18 ± 0.05^B^	0.29 ± 0.02^ab^	0.70 ± 0.18^A^	3.17 ± 0.30^a^*	0.22 ± 0.12^B^	0.08 ± 0.03^b^	1.33 ± 0.11^AB^	1.18 ± 0.08^a^
Dep	− 0.09 ± 0.01^A^	− 0.30 ± 0.02^a^*	− 0.85 ± 0.03^A^	− 0.51 ± 0.10^ab^	− 3.62 ± 0.08^B^	− 6.62 ± 0.12^b^**	− 0.54 ± 0.10^AB^	− 0.89 ± 0.09^a^
App	0.37 ± 0.09^BC^	0.52 ± 0.07^b^	1.21 ± 0.40^AB^	12.91 ± 0.72^a^**	0.31 ± 0.10^C^	0.08 ± 0.02^b^	4.16 ± 0.07^AB^	2.46 ± 0.28^a^*
Net	0.28 ± 0.10^BC^	0.21 ± 0.11^a^	0.36 ± 0.09^A^	12.41 ± 0.82^a^**	− 3.32 ± 0.16^C^	− 6.55 ± 0.15^b^**	3.62 ± 0.17^AB^	1.57 ± 0.08^a^*
Turn	0.46 ± 0.09^C^	0.82 ± 0.07^b^*	2.06 ± 0.44^AB^	2.06 ± 0.44^ab^**	3.91 ± 0.07^BC^	6.71 ± 0.09^a^*	4.70 ± 0.04^AB^	3.34 ± 0.19^ab^*
**Semi- EAAs**								
Arg	− 0.07 ± 0.01^A^	− 0.19 ± 0.03^a^*	− 0.6 ± 0.07^AB^	− 0.9 ± 0.03^b^	− 0.80 ± 0.06^B^	− 1.60 ± 0.03^b^*	− 0.29 ± 0.03^AB^	− 0.67 ± 0.08^ab^*
Gly	0.04 ± 0.01^A^	− 0.11 ± 0.02^a^*	− 0.83 ± 0.10^AB^	− 1.69 ± 0.05^b^*	− 2.76 ± 0.46^B^	− 4.11 ± 0.18^c^	− 0.51 ± 0.03^AB^	− 1.40 ± 0.12^bc^*
Tyr	− 0.05 ± 0.01^A^	− 0.13 ± 0.02^a^*	− 0.41 ± 0.01^AB^	− 0.28 ± 0.02^ab^	− 0.79 ± 0.13^B^	− 1.49 ± 0.15^b^*	− 0.38 ± 0.05^AB^	− 0.58 ± 0.06^ab^
Ser	0.0 ± 0.0^A^	0.03 ± 0.01a	− 0.58 ± 0.04^AB^	− 1.12 ± 0.1^b^*	− 1.12 ± 0.26^B^	− 1.92 ± 0.11^c^	− 0.56 ± 0.04^AB^	− 0.79 ± 0.07^bc^
Gln	0.0 ± 0.00^A^	− 0.14 ± 0.02^a^*	− 0.25 ± 0.01^AB^	0.0 ± 0.0^a^	− 2.34 ± 0.65^B^	− 4.01 ± 0.11^b^	0.58 ± 0.01^A^	− 0.25 ± 0.03^ab^*
Dep	− 0.12 ± 0.01^A^	− 0.61 ± 0.09^a^*	− 2.67 ± 0.23^AB^	− 4.0 ± 0.13^bc^*	− 7.81 ± 1.68^C^	− 11.56 ± 0.83^c^	− 1.76 ± 0.18^AB^	− 3.68 ± 0.28^b^*
App	0.04 ± 0.01	0.03 ± 0.02	0.0 ± 0.0	0.003 ± 0.002	0.0 ± 0.00	0.0 ± 0.00	0.58 ± 0.01	− 0.0 ± 0.0
Net	− 0.08 ± 0.02^A^	− 0.58 ± 0.07^a^*	− 2.67 ± 0.23^AB^	− 4.0 ± 0.13^bc^*	− 7.81 ± 1.68^C^	− 11.56 ± 0.83^c^	− 1.18 ± 0.18^AB^	− 3.68 ± 0.28^b^*
Turn	0.17 ± 0.01^C^	0.65 ± 0.11^a^*	2.67 ± 0.23^AB^	4.0 ± 0.13^ab^*	7.81 ± 1.68^A^	11.56 ± 0.83^c^	2.34 ± 0.19^BC^	3.68 ± 0.28^b^*
**Total AAs**								
Dep	− 0.43 ± 0.03A	− 1.46 ± 0.04^a^*	− 6.46 ± 0.25^AB^	− 7.77 ± 0.25^bc^	− 18.06 ± 3.62^C^	− 31.53 ± 1.51^c^*	− 4.16 ± 0.59^AB^	− 8.35 ± 0.20^b^*
App	0.75 ± 0.12^BC^	1.24 ± 0.07^ab^	1.46 ± 0.05^AB^	14.09 ± 0.64^a^**	0.60 ± 0.14^C^	0.08 ± 0.03^b^	5.44 ± 0.08^AB^	2.68 ± 0.22^a^*
Net	0.32 ± 0.15^B^	− 0.22 ± 0.01^a^	− 5.0 ± 0.16^AB^	6.31 ± 0.29^a^**	− 17.46 ± 1.90^C^	− 31.46 ± 1.54^c^*	1.28 ± 0.50^AB^	− 5.66 ± 0.51^b^*
Turn	1.18 ± 0.09^B^	2.71 ± 0.23^c^*	7.92 ± 0.25^BC^	21.86 ± 0.39^a^**	18.66 ± 1.35^A^	31.61 ± 1.47^a^*	9.59 ± 0.66^AB^	11.03 ± 0.11^ab^

Differences between the concentration of AA in the test medium (after 24 h incubation) and the fresh medium were used to calculate the depletion or appearance of each AA. Negative and positive values indicate the “depletion (Dep)” and “appearance (App)” of AAs, respectively. The “total net balance (Net)” of all AAs was calculated by subtracting “total depletion” from “total appearance”. In addition, the “total turnover (Turn)” of all AAs was calculated by the sum of “total depletion” and “total appearance”. The data were obtained from 6 measurements. Asterisks indicate significant differences between cleaved *vs.* non-cleaved groups within each experimental treatment; data was normally distributed and analyzed using t-test with a Bonferroni correction (*: *p* < 0.05; **: *p* < 0.01; ***: *p* < 0.001). Different capital or small letters indicate significant differences among cleaved or non-cleaved groups across all experimental treatments, respectively (*p* < 0.05, one-way ANOVA followed by Tukey's multiple comparison test, n = 6). Data are means ± S.E.M. ^**#**^indicates the percentage of cleaved or non-cleaved COCs in relation to the initial number of COCs cultured in the maturation medium.

In the EGF group, the non-cleaved COCs consumed significantly more Met (*p* < 0.05), Trp (*p* < 0.05), Lys (*p* < 0.01), Glu (*p* < 0.01), Gly (*p* < 0.05) and Ser (*p* < 0.05) and produced more Ala (*p* < 0.01) and Orn (*p* < 0.05) compared to the cleaved COCs. Moreover, the total depletion, net balance and turnover of all AAs in the EGF group (Table 1) was higher in the non-cleaved COCs than in the cleaved COCs (*p* < 0.01).

In the urea group, the non-cleaved COCs consumed significantly more Ile, Lys, Cit, Glu, Asp, Arg, and Tyr and had a higher total depletion, net balance and turnover of all AAs (*p* < 0.05) compared to the cleaved COCs (Table 1).

In the EGF-urea (EU) group, Met, Ile, Lys, Glu, Asp, Arg, Gly, and Gln depletion was higher in the non-cleaved COCs than in the cleaved COCs (*p* < 0.05). Non-cleaved COCs displayed a higher total depletion and net balance of all AAs and produced a lower amount of Ala compared to cleaved COCs (*p* < 0.05, Table 1).

### Univariate analysis for the evaluation of cleavage and depletion/appearance of AAs by COCs matured individually

In the individual-IVM system, urea abrogated the EGF-induced the percentage of COCs reached to the cleavage stage. It was found that the percentage of cleavage decreased from 66.7% (the EGF group) to 44.8% (the EGF + urea group, approximately 66%, Table 1).

Cleaved COCs in the urea group significantly consumed more Thr, Val, Leu, Asp, Arg, Gly, Tyr, Ser and Gln and produced lower amount of Ala than the cleaved COCs in the control group (*p* < 0.05). Cleaved COCs in the urea group had a higher total depletion, net balance and turnover of all AAs compared to the cleaved COCs in the control group (*p* < 0.01). Generally, COCs matured in the presence of urea consumed higher quantities of AAs (Table 1).

Non-cleaved COCs of the urea group significantly consumed more of all AAs compared to the non-cleaved COCs of the control group (*p* < 0.01). The total depletion, net balance and turnover of all AAs were higher in the non-cleaved COCs in the urea group compared to the non-cleaved COCs in the control group (*p* < 0.01). Numerically, non-cleaved COCs in the urea group consumed more AAs than other non-cleaved groups (Table 1).

### *N*-acetyl-l-cysteine completely abrogates urea-induced depletion of AAs

Data showed that the NAC completely abrogated the urea-induced depletion of AAs (*p* < 0.05, Fig. 4c).

### Bivariate analysis to assess the association between AAs and the developmental competence of COCs matured individually or in groups

**Figure 5:**
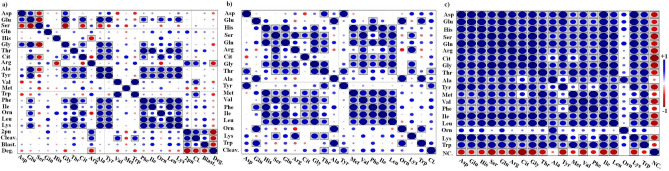
**(a) **Heatmap of the pairwise correlation of amino acids and developmental competence of COCs matured in** (a) **a group-IVM culture;** (b) **an individual-IVM culture (cleaved COCs); and** (c) **an individual-IVM culture (non-cleaved COCs). The Anderson–Darling test showed that the group-IVM culture data was not normally distributed and that the individual-IVM culture data was normally distributed. Therefore, the Pearson correlation and Kendall rank correlation was used for normal and log-transformed data, respectively. Boxed squares mean a significant correlation (*p* < 0.05). The Bonferroni correction was used to test the significance of the correlations in the correlation matrix. Red and blue circles display negative and positive correlations, respectively. Scale bar on the right side of the pairwise heatmap shows the correlation between the factors. *Deg* percentage of oocyte degeneration, *Cleav* cleavage stage, *Blast* blastocyst stage, *2PN* two-pronuclear zygote, *Cl* cleaved COCs, *NC* non-cleaved COCs.

Using group-matured COC data, the Kendall correlation showed a correlation between 2-PN and Arg (R = 0.73, *p* < 0.05, Fig. 5a and Supplementary Fig. 2). It was also found that the percentage of COCs reached the cleavage stage (2–16 cell stage) was correlated with Arg (R = 0.58, *p* < 0.05). The Kendall correlation analysis did not find any significant correlation between the blastocyst formation and AAs. In addition, Arg (R = 0.40, *p* = 0.07), Leu (R = 0.34, *p* = 0.12) and Thr (R = 0.37, *p* = 0.09) tended to be positively associated with the blastocyst formation. Moreover, none of the AAs were associated with the blastocyst formation in each experimental treatment. The pairwise comparison heatmap (Fig. 5a) exhibited that oocyte degeneration (by day 3 post-insemination) was negatively correlated with Arg (R = − 0.62, *p* < 0.05).

The Pearson correlation revealed that the percentage of cleaved COCs matured individually (relative to the initial cultured COCs) was correlated with Tyr (R = 0.71, *p* < 0.05) and Thr (R = 0.58, *p* < 0.05). Moreover, Glu (R = 0.57, *p* = 0.06), Cit (R = 0.45, *p* = 0.14), Val (R = 0.53, *p* = 0.08), Trp (R = 0.49, *p* = 0.10) and Phe (R = 0.55, *p* = 0.07) tended to be correlated with cleaved COCs (Fig. 5b and Supplementary Fig. 3).

Pearson analysis showed that the percentage of non-cleaved COCs matured individually (relative to the initial cultured COCs) was negatively correlated with the turnover of all AAs. Data showed a correlation between the percentage of non-cleaved COCs and the turnover of Asp (R = − 0.71, *p* < 0.01), Glu (R = − 0.60, *p* < 0.04), His (R = − 0.65, *p* < 0.02), Ser (R = − 0.82, *p* < 0.001), Gln (R = − 0.67, *p* < 0.02), Arg (R = − 0.77, *p* < 0.003), Cit (R = − 0.92, *p* < 0.001), Gly (R = − 0.81, *p* < 0.001), Thr (R = − 0.67, *p* < 0.02), Tyr (R = − 0.76, *p* < 0.01), Val (R = − 0.75, *p* < 0.01), Ile (R = − 0.77, *p* < 0.01), Leu (R = − 0.75, *p* < 0.01) and Lys (R = − 0.71, *p* < 0.01). In addition, Met (R = − 0.51, *p* < 0.09), Phe (R = − 0.54, *p* < 0.07) and Trp (R = − 0.49, *p* < 0.10) tended to be associated with non-cleaved COCs (Fig. 5c and Supplementary Fig. 4).

### The heatmap analysis supports the univariate analysis findings

**Figure 6:**
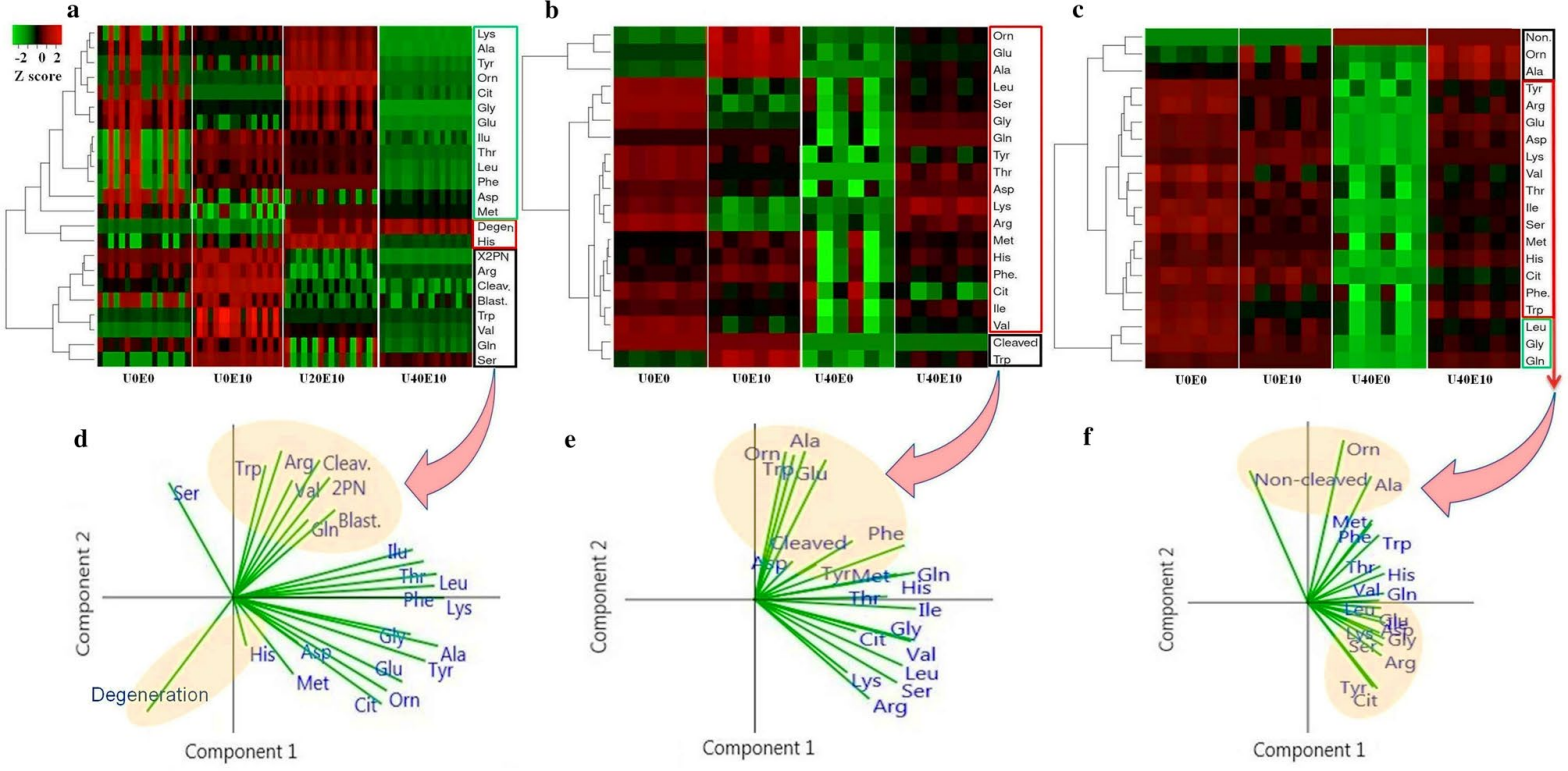
**(a–c)** The hierarchical cluster and the heatmap of the depletion/appearance of amino acids and the developmental competence of COCs. COCs were matured in (**a**) a group-IVM culture; (**b**) an individual-IVM culture (cleaved COCs); and (**c)** an individual-IVM culture (non-cleaved COCs) under different treatments. Heatmap was generated by “Heatmapper web tool”. Kendall’s tau was used for non-parametric data derived from the group-IVM culture and Pearson was used for normal data obtained from the individual-IVM culture. Green represents higher depletion (or lower appearance) of AAs or lower rates of other factors, i.e., oocyte competence. Red represents a greater appearance of AAs or higher percentage of oocyte competence. The color intensity indicates the variation of the values in the color scale on the left side of the heatmap. **(a–c**) Different clusters are placed in different color boxes. **(d–f) **Biplot generated from the principal component analysis (PCA) of factors, including amino acid data and the developmental competence of COCs. COCs were matured in (**d**) a group-IVM culture; (**e**) an individual-IVM culture (cleaved COCs); and (**f**) an individual-IVM culture (non-cleaved COCs). Vectors with close angles (< 45°) imply a strong correlation, perpendicular vectors indicate no correlation, and vectors in opposite directions (approaching 180°) indicate a negative correlation. *Degen* percentage of oocyte degeneration, *Cleav* percentage of cleavage, *Blast* percentage of blastocyst, *2PN* two-pronuclear zygote, *Cleaved* cleaved COCs, *Non* non-cleaved COCs.

The color intensity of the heatmap represents the depletion and appearance of the AAs and the developmental competence of the individual or group matured COCs (Fig. 6a–c). Green represents a higher depletion (or lower appearance) of AAs or a lower percentage of other variables, i.e., oocyte competence. Red represents a higher appearance of AAs or higher levels of oocyte competence.

The heatmap analysis showed that, in the presence of EGF and a high level of urea, the turnover of all AAs (except Ser) by COCs (matured in groups) was higher than other experimental treatments. In addition, the heatmap analysis verified the results of the ANOVA test in such a way that oocyte degeneration increased in the presence of EGF and a high level of urea during IVM, but the percentage of COCs reached the cleavage, 2-PN and blastocyst stages decreased (Fig. 6a).

The heatmap analysis showed that when COCs were matured individually, the depletion of AAs by COCs was greater in the urea group (both cleaved and non cleaved COCs) than in other treatments whereas the non-cleaved COCs depleted substantially more AAs than the cleaved COCs (Fig. 6b,c). In the case of individual-IVM culture, EGF abrogated the urea-increased depletion of AAs by COCs, but this effect was not achieved in the group-IVM culture (Fig. 6b,c).

### A hierarchical cluster analysis, a multivariate approach, to classify the related variables

Using the Kendall and Pearson distance metric and a complete-linkage clustering method, cluster analysis was done to classify factors that are close together (Fig. 6a–c). Since the data from COCs matured in groups were not normally distributed, we used Kendall distance metric as a non-parametric test. The Pearson distance metric was used to identify the normal data obtained from individually matured COCs.

In the case of the group-IVM culture, the hierarchical cluster analysis revealed the similarity (lowest distance) between Arg and the percentage of 2PN and cleavage, since they were linked together (black box in Fig. 6a). Percentage of 2PN, cleavage, blastocyst formation, Arg, Trp, Val, Ser, and Gln all together formed a cluster (black box in Fig. 6a). Oocyte degeneration and His formed a distinct cluster together (red box in Fig. 6a).

In the case of the individual-IVM culture, the hierarchical dendrogram showed a correlation between the percentage of the cleaved COCs and Trp (black box in Fig. 6b). The hierarchical dendrogram showed the similarity between Orn, Ala and percentage of non-cleaved COCs (black box in Fig. 6c).

### The principal component analysis, a multivariate analysis, to assess the association between AAs and the developmental competence of COCs

The principal component analysis (PCA) was performed in order to reduce the broad range of variables (high dimensional data) and to find a concurrent association between AAs and oocyte competence. Data of AAs depletion/appearance by COCs matured individually or in groups were analyzed using PAST software. The first three principal axis factors accounted for a sufficient amount of total variation (77.8%, 90.6%, and 96.4% found for data of COCs cleaved and non-cleaved in the individual-IVM culture or in group-IVM culture, respectively, Supplementary Table 2).

In the case of group-IVM culture, PCA identified a strong positive correlation between Trp, Arg, Gln, Ile, Val and the percentage of COCs reached the 2PN, cleavage and blastocyst stages (directional vectors < 45°, Fig. 6d). There was a strong negative correlation between Trp, Arg, Gln, Ile, Val and oocyte degeneration (directional vectors approaching 180°). The PCA showed a strong positive association between His and oocyte degeneration (Fig. 6d).

In the case of the individual-IVM culture, PCA identified a strong positive correlation between Orn, Trp, Ala, Glu, Asp, Phe, Tyr, Met and the percentage of cleaved COCs (directional vectors < 45°, Fig. 6e). The data showed a strong positive correlation between Orn, Ala, and the percentage of non-cleaved COCs (Fig. 6f). Moreover, PCA identified a strong negative correlation between Cit, Tyr, Arg, Ser, Gly, Lys,Asp, Glu, Leu and the percentage of non-cleaved COCs (Fig. 6f). Such observation of the PCA confirmed the results of the hierarchical cluster analysis (Fig. 6a–c).

### Sub-network enrichment analysis describes the correlation between Arg and the incidence of high developmental competence

**Figure 7:**
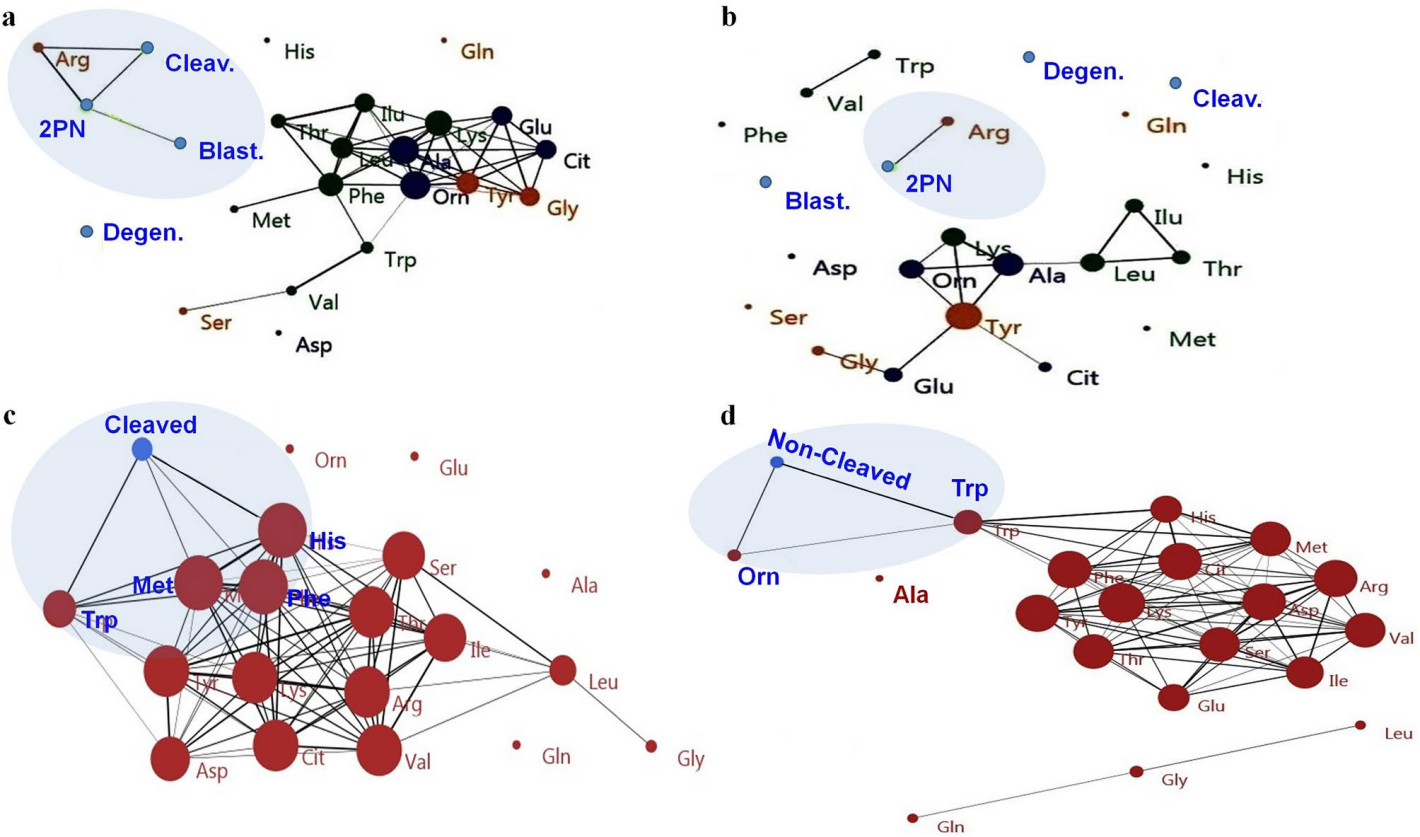
Correlation-based network analysis of amino acids (AAs) and developmental competence of COCs. COCs were matured in (**a,b**) a group-IVM culture; (**c**) an individual-IVM culture (cleaved COCs); and (**d**) an individual-IVM culture (non-cleaved COCs). All AAs and parameters reflecting developmental competence are defined by circles within the network. Network analysis and visualization was carried out using PAST and Fruchterman-Reingold algorithm as a force-directed layout algorithm. The Rho correlation thresholds of **(a)** 80%, **(b)** 90% were selected to identify the connections between edges and nodes in terms of the high percentage of developmental competence of COCs matured in the group-IVM culture. The Pearson correlation thresholds of **(c)** 72% and **(d)** 82% were selected to identify the connections between edges and nodes in terms of the cleaved or non-cleaved COCs in the individual-IVM culture, respectively. Nodes represent AAs and parameters related to oocyte competence. Edges are the interactions between all factors. The size of the nodes and edges refers to the coefficient of clustering and the coefficient of correlation, respectively. Small nodes and thin edges refer to small values.

The nodes of the enriched sub-modules indicate AAs and parameters representing developmental competence (Fig. 7). The node size reflects the degree of connectivity of the node (i.e., AAs and parameters representing developmental competence) and the edges show the relationship between the two variables. The thicker edges reveal higher correlations between variables. Nodes with more links are close to each other.

In the case of the group-IVM culture, the amino acid-developmental competence interaction networks with the cutoff point > 80% showed that Arg was associated with the percentage of COCs reached the 2PN, cleavage and blastocyst stages (Fig. 7a). More specifically, when the cutoff point increased from 80 to 90%, only Arg had an association with the percentage of COCs reached the 2PN stage (Fig. 7b).

In the case of individual-IVM culture, the amino acid-cleavage interaction networks with the cutoff point > 82% showed that the cleaved COCs were associated with Trp, Met, His and Phe (Fig. 7c). In addition, a network analysis > 72% of the cutoff point showed an association between Trp, Orn, and non-cleaved COCs (Fig. 7d).

### The receiver operator characteristic (ROC) curve analysis

To be more specific, the ROC curve study was performed to determine the predictive power of variables (i.e., AAs) and to calculate the optimal cutoff for the positive outcome prediction. In the case of group-IVM culture, AAs were divided into two separate groups based on IVF outcomes (i.e., high 2-PN percentage > 80% *vs*. low 2-PN percentage; high percentage of cleavage > 80% *vs*. low percentage of cleavage; high percentage of blastocyst formation > 30% *vs*. low percentage of blastocyst formation; and low percentage of oocyte degeneration < 15% *vs*. high percentage of oocyte degeneration). In the case of individual-IVM culture, the AAs were divided into two classes based on the incidence of cleavage as follows: the cleaved *vs*. the non-cleaved COCs. The AUC, *P*-value, optimal cutoff point, sensitivity, specificity, positive/negative predictive values and positive/negative likelihood ratios obtained for the significant AUC are shown in Table 2. The results showed that the positive likelihood ratio (LR) of all factors was much more than 1.0. However, the negative LR of all factors was less than 1.0, suggesting that there was an increased likelihood of a high percentage of developmental competence^26^ (Table 2).

**Table 2:** Rank of amino acids turnover (depletion/appearance) based on their discriminating power (AUC) from ROC curve analysis.

	AUC	*p*-value	Optimal cutoff point	Sensitivity	Specificity	Positive predictive value	Negative predictive value	Positive likelihood ratio (LR)	Negative likelihood ratio (LR)	AUC rank
**The proportion of 2PN (the group-IVM system)**										
Arg	1.00	< 0.000001	− 0.60	100	100	100	100	Inf	0.0	1
Gln	0.75	0.09	− 8.90	66.7	100	100	75.0	Inf	0.33	2
**The proportion of oocyte degeneration, by day 3 post-insemination (the group-IVM system)**										
Arg	0.91	< 0.00001	− 0.60	85.7	100	100	83.3	Inf	0.14	1
Gln	0.74	0.12	− 8.90	57.1	100	100	62.5	Inf	0.43	2
**The percentage of cleavage, 2–16 cell stage (the group-IVM system)**										
Val	1.0	0.000001	1.16	100	100	100	100	Inf	0.0	1
App. EAAs	1.0	0.000001	7.14	100	100	100	100	Inf	0.0	2
Turn. EAAs	1.0	0.000001	7.14	100	100	100	75.0	Inf	0.0	3
Arg	0.96	0.000003	− 0.17	88.9	100	100	75.0	Inf	0.11	4
Ser	0.93	0.000005	− 1.65	88.9	100	100	75.0	Inf	0.11	5
Trp	0.89	0.0001	0.20	66.7	100	100	50.0	Inf	0.33	6
Net. EAAs	0.89	0.0001	7.14	66.7	100	100	50.0	Inf	0.33	7
Thr	0.89	0.0001	1.32	88.9	100	100	75.0	Inf	0.11	8
Dep. Total	0.89	0.0001	− 9.39	88.9	100	100	75.0	Inf	0.11	9
Net. Total	0.85	0.003	19.43	100	66.7	90.0	100	3.0	0.0	10
Dep. Semi	0.85	0.003	− 11.08	77.8	100	100	60.0	Inf	0.22	11
Net. Semi	0.85	0.003	− 6.40	77.8	100	100	60.0	Inf	0.22	12
Ile	0.82	0.02	0.32	66.7	100	100	50	Inf	0.33	13
**The percentage of blastocyst formation, day 7 post-insemination (the group-IVM system)**										
Arg	0.84	0.003	− 0.60	75.0	100	100	66.7	Inf	0.25	1
Leu	0.84	0.003	0.97	87.5	75.0	87.5	75.0	3.5	0.17	2
Gln	0.84	0.003	− 8.23	75.0	100	100	66.7	Inf	0.25	3
Dep. Semi	0.84	0.003	− 11.18	75.0	100	100	66.7	Inf	0.25	4
Ile	0.81	0.01	0.57	100	75.0	88.9	100	4.0	0.0	5
Thr	0.78	0.04	1.35	100	75.0	88.9	100	4.0	0.0	6
**The proportion of the cleaved COCs, 2–16 cell stage (the individual-IVM system)**										
Glu	0.76	0.0001	− 0.03	75.0	83.3	81.8	76.9	4.5	0.31	1
Ala	0.72	0.003	2.0	50.0	100	100	66.7	Inf	0.52	2
Ile	0.71	0.003	− 0.4	83.3	75.0	76.9	81.8	3.3	0.21	3
Trp	0.70	0.005	0.35	41.7	100	100	63.2	Inf	0.51	4
Met	0.69	0.009	− 0.01	83.3	91.7	90.9	84.6	10.0	0.20	5
Orn	0.68	0.01	1.32	41.7	91.7	83.3	61.1	5.0	0.61	6
Phe	0.67	0.03	− 0.08	75.0	66.7	69.2	72.7	2.3	0.37	7
Tyr	0.66	0.03	− 0.40	75.0	66.7	69.2	72.7	2.3	0.37	8
Arg	0.65	0.05	− 0.18	100	75.0	57.1	100	1.3	0.0	9

The ROC curve study was performed to determine the predictive power of factors (i.e., AAs) and to measure the optimal cutoff point for the positive outcome prediction. The AAs were divided into two separate groups based on the IVF outcomes of the group-IVM system (i.e., high 2PN percentage > 80%, high percentage of cleavage > 80%, high percentage of blastocyst formation > 30%; low percentage of oocyte degeneration < 15%) and the individual-IVM culture (i.e., incidence of cleavage vs. absence of cleavage).

*Dep* depletion, *Net* net balance, *App* appearance, *Turn* turnover, *Total* all amino acids, *Non* non-essential amino acids, *Semi* semi-essential amino acids, *EAA* essential amino acids, *2PN* two-pronuclear zygote.

In the case of group-IVM culture, the ROC curve analysis for predicting the existence of a high 2PN percentage > 80% showed that Arg had the best predictive power with a significant area under the curve (AUC) of 1.00, a sensitivity of 100% and a specificity of 100%. Moreover, the depletion level of Arg > -0.60 pmol/h was the best optimal cutoff point to predict a high 2PN percentage (Table 2, Supplementary Fig. 5a).

The AUC curve analysis was also used to determine the diagnostic value of AAs by COCs matured in groups for predicting the presence of low oocyte degeneration (i.e., < 15%). We observed that Arg had the highest predictive performance with a strong AUC of 0.91, a sensitivity of 85.7% and a specificity of 100%. The ROC analysis defined the depletion levels of Arg > − 0.60 pmol/h as the optimal cutoff for estimating the incidence of low oocyte degeneration (Table 2, Supplementary Fig. 5b).

Val (AUC = 1.00), Arg (AUC = 0.96), Ser (AUC = 0.93), Trp (AUC = 0.89), Thr (AUC = 0.89), Ile (AUC = 0.92) and the total turnover (AUC = 1.00), total appearance (AUC = 1.00) and total net balance (AUC = 0.89) of EAAs were the factors with the highest AUC for detecting a high percentage of cleavage > 80%. Moreover, the total depletion (AUC = 0.89) and net balance (AUC = 0.85) of all AAs and total depletion (AUC = 0.85) and net balance (AUC = 0.85) of semi-EAAs showed the best AUC for predicting a high percentage of cleavage > 80%. Furthermore, the optimal cutoff points for predicting a high percentage of cleavage were > 1.16, > -0.17, > -1.65, > 0.2, > 1.32, and > 0.32 pmol/h for Val, Arg, Ser, Trp, Thr and Ile, respectively (Table 2 and Supplementary Fig. 5c).

Evaluation of the ROC curve for the presence of a high percentage of blastocyst formation (i.e., > 80%) showed that Arg, Leu, Gln, Ile, and Thr had the highest predictive ability performance with substantial AUC values of 0.84, 0.84, 0.84, 0.81, and 0.74, respectively. Moreover, the optimal cutoff for predicting a high percentage of blastocysts formation was estimated as > − 0.60, > 0.97, > − 8.23, > 0.57, and > 1.35 pmol/h for Arg, Leu, Gln, Ile, and Thr, respectively (Table 2, Supplementary Fig. 5d).

In the case of individual-IVM culture, the ROC curve analysis for predicting the incidence of cleavage showed that Glu, Ala, Ile, Trp, Met, Orn, Phe, Tyr, and Arg had the best predictive power with a significant area under the curve (AUC) of 0.76, 0.72, 0.71, 0.70, 0.69, 0.68, 0.67, 0.66, and 0.65, respectively. Data showed that depletion levels of Glu > − 0.03, Ala < 2.0, Ile > − 0.4, Trp > 0.35, Met > − 0.01, Orn > 1.32, Phe > − 0.08, Tyr > − 0.4 and Arg > − 0.18 pmol/h can be used as the best optimal cutoff points for predicting the incidence of cleavage (Table 2, Supplementary Fig. 5e).

### In silico molecular docking study reveals the molecular association of urea with DNA strains, EGF, EGFR and aquaporin (AQP) 3

It was evident from the ANOVA analysis that, in the presence of urea, oocyte degeneration increased and EGF-induced cleavage formation was abolished. These findings encouraged us to investigate whether urea interacts with DNA; this may help us to explain some of the increased percentage of oocyte degeneration found in this study. Next, we sought to find the feasibility of association between urea and EGF/EGFR; this was achieved to determine how urea would bind with these molecules. The potential interaction between urea and these molecules will allow us to better understand the interference of urea in the EGF-EGFR binding. In order to study changes in the energy binding of urea to DNA, EGF, EGRFR, and AQP3, with an increase in number of urea molecules, molecular docking was conducted 20, 12, 12 and 12 consecutive times, respectively. Indeed, each time the previous docked EGFR-urea, EGF-urea, or DNA-urea complex was used as a receptor for the next docking run under the same conditions.

**Figure 8:**
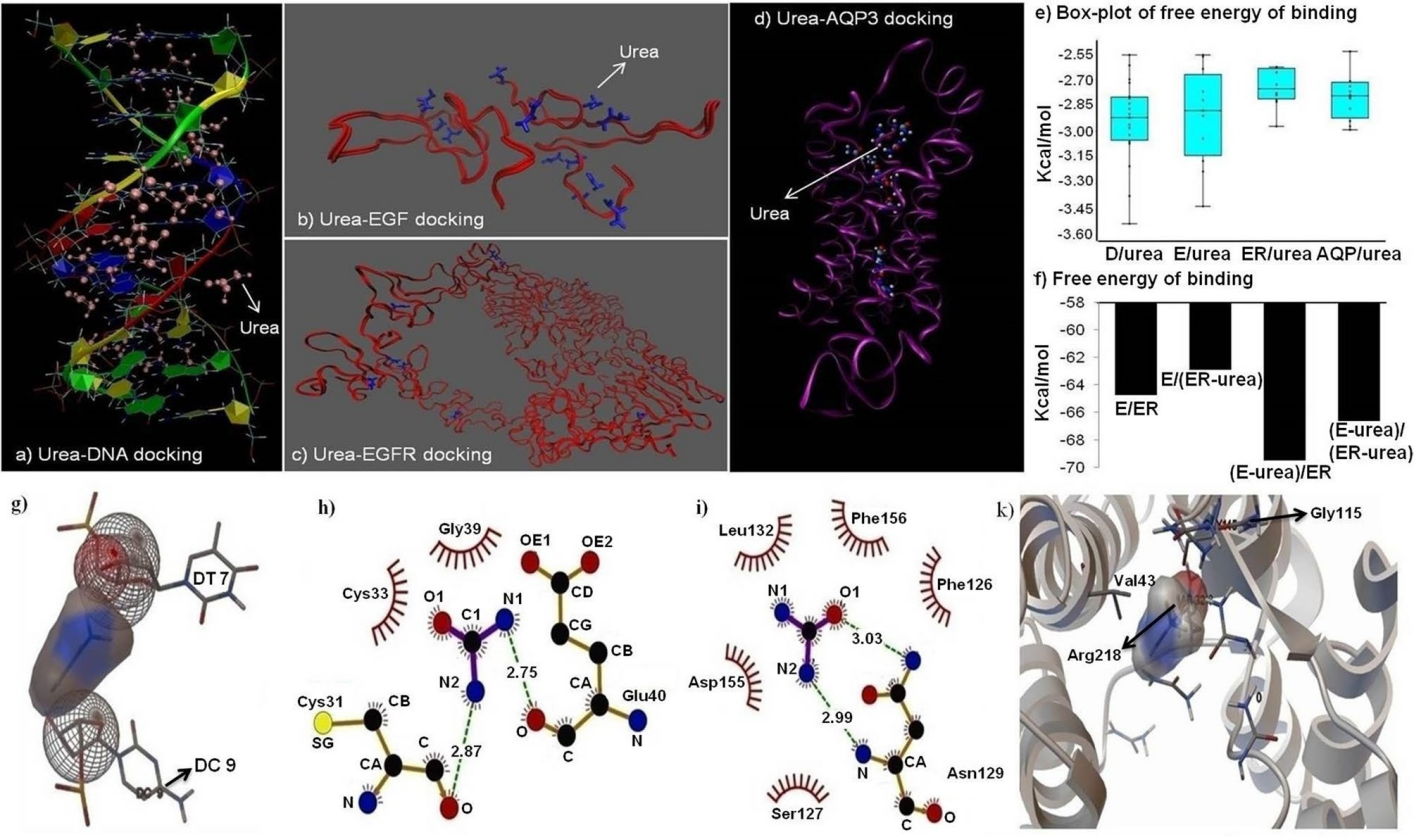
In silico study of urea binding to **(a)** DNA, **(b)** EGF, **(c)** EGFR, and **(d)** AQP3. Optimized urea was docked with these molecules by AutoDock 4. 2. Using Lamarkian Genetic Algorithm, molecular docking was performed to determine the binding free energy and the orientation of urea in its interaction with these molecules. The crystal form of the extracellular domain of EGFR (PDB ID: 3NJP), EGF (1jl9), and DNA (1 BNA) was taken from the Protein Data Bank. Computer modeling on AQP3 (ID: Q08DE6) was performed by providing amino acid sequences from the Universal Protein Resource (UniProt) database, followed by predicting a 3-D structure on the I-TASSER server. To assess the urea binding behavior, molecular docking was conducted at 20, 12, 12, and 12 sequential times for urea-DNA, urea-EGF, urea-EGFR, and urea-AQP3, respectively. Indeed, each time the previous docked urea-receptor complex was used as a receptor for the next docking run under the same conditions**.** These images (**a–d**) were produced by the VMD molecular visualization and analysis software (V. 1.9.2.). **(e)** Box plot to display the energy binding of urea to DNA, EGF, EGFR, and AQP3. **(f)** In order to investigate how the energy binding of EGF with EGFR shifts in the presence of urea, molecular docking was performed in four phases as follows: phase I: EGF/EGFR; phase II: (EGF-urea complex)/EGFR; phase III: EGF/(EGFR-urea complex); and phase IV: (urea-EGF complex)/(EGFR-urea complex). The EGF-urea and EGFR-urea complexes obtained from the preceding step were used in these phases. In the panels (**g**) and (**k**), the ball represents the van der waals volume interactions. These images (**g,k**) were produced by the MGLTools software (V. 1.5.6.). Hydrogen bonds are shown in panels (**h**) and (**i**) by dashed green lines. Also, the distance between the receptor and the donor atoms is shown in the middle. Hydrophobic contacts are represented by arcs with spokes which radiate towards urea molecules. LIGPLOT software (V. 2.2.) was used to produce images and visualize hydrophilic interactions, hydrogen bonding, and hydrophobic contacts between urea and EGF (**h**) and EGFR (**i**). E, EGF; D, DNA; ER, EGFR; and AQP, aquaporin 3.

Molecular docking showed that free energy of binding was negative for all urea interactions with DNA, EGF, extracellular domain of EGFR, and AQP3 (Fig. 8a–e). Box-plot analysis showed that urea had favorable binding activity against DNA strains (− 2.94 kcal/mol, 95% CI: − 3.05, − 2.83), EGF (− 2.91 kcal/mol: CI 95%: − 3.07, − 2.74), extracellular domain of EGFR (− 2.74 kcal/mol, 95% CI: − 2.82, − 2.67), and AQP3 (− 2.79 kcal/mol, 95% CI: − 2.88, − 2.70; Fig. 8e). It suggested, therefore, that urea had an increased affinity for DNA and EGF.

Next, in the presence of urea, molecular docking was performed again to compare the change in the free energy binding of EGF-EGFR complex. To this aim, the previous docked EGFR-urea or EGF-urea complex was used as a receptor for these docking processes. The total interaction energy(E_tot_) was − 64.76, − 63.0, − 69.5, and − 66.7 kcal/mol for: (I) EGF docking with the extracellular domain of EGFR; (II) EGF docking with the extracellular domain of EGFR-urea complex; (III) EGF-urea complex docking with the extracellular domain of EGFR; and (IV) EGF-urea complex docking with the extracellular domain of EGFR-urea complex, respectively (Fig. 8f). This finding suggested the strongest interactive impact of urea on the EGFR molecule.

The molecular docking analysis showed the interaction of urea with DNA bases (e.g., thymine and cytosine; Fig. 8g). Using LIGPLOT software, we found hydrogen bonds and non-covalent contacts between urea and some EGF residues and the extracellular domain of EGFR (Fig. 8h,i). The nitrogen atoms in urea molecules were mainly involved in hydrophobic interactions with the residues, such as Cys33, Gly39, Leu132 and Phe156. Furthermore, there are some hydrogen bonds between hydrogen atoms of urea molecules and oxygen atoms of residues (red arcs in Fig. 8h,i). In addition, the LIGPLOT showed the existence of hydrogen bonds between urea and some residues of EGF and EGFR, such as Cys31, Asn129, and Glu40 (green dashes; Fig. 8h,i). It was observed that there were several van der waals volume interactions between urea and AQP3, like Val43, Gly115 and Arg218 (Fig. 8k).

## Discussion

In this study, the conditions of IVM modified the relationship between the metabolism of AAs and the developmental competence of COCs. Data showed that, in response to the presence of EGF and urea in the group-IVM culture, COCs released lower quantities of Thr, Phe, Val, Leu, Trp, Gly and Ala, consumed more Ile, Lys, Gln and Arg, and showed lower developmental competence. Moreover, the depletion of AAs was the highest in urea-incubated COCs with greater depletion in non-cleaved COCs than in cleaved COCs using an individual-IVM culture. Recently, we observed a greater depletion of AAs and a decrease in oocyte competence in the presence of high urea levels in the group-IVM system^17^. Similarly, Hemmings et al. reported that higher AA depletion is associated with a decrease in the developmental competence of oocytes in humans and bovines^18,19^. A growing body of evidence suggests that non-cleaved oocytes consume (deplete) more AAs for DNA repair and prevention of degeneration^18,27^. Greene et al. reported that the addition of Arg to the cell culture reduced the fragmentation of DNA in human endometrial cells^28^. It seems that COCs may increase the consumption of AAs to meet their needs in order to cope with the negative effects of urea as a cell-stressor^18,27^.

As mentioned above, more AAs were depleted by COCs in the presence of EGF and a high level of urea using the group-IVM system (Fig. 6a). In view of the fact that the EGF may inhibit AAs transport^23^, it is proposed that the increased depletion of AAs by COCs can result from the inhibiting action of urea on the EGF/EGFR system^9–11^. COCs were matured in the presence of NAC in order to test the potential interactive effect of urea on AAs metabolism via EGFR. It has been reported that NAC may inhibit the activation of EGFR^29^. Moreover, urea signaling has been shown to be sensitive to NAC^29^. Current data showed that NAC completely abrogated the urea-induced depletion of AAs by COCs. This meant that urea could use the EGFR system to increase the consumption of AAs by COCs. In addition, as shown by the present in silico molecular docking, both EGF and EGFR could be bound by urea, suggesting an interference role for urea in the EGF/EGFR system. Moreover, molecular docking showed that E_tot_ for the EGF/EGFR-urea complex (− 62.96 kcal/mol) was the lowest compared to the EGF/EGFR complex (− 64.76 kcal/mol) and the EGF-urea complex/EGFR (− 69.49 kcal/mol). It seems that, in the presence of urea, the extracellular domain of EGFR may have a lower affinity to the EGF. This indicated that urea interfered with the interaction between EGF and EGFR. Therefore, urea can reduce the inhibitory effect of EGF on AAs transport, resulting in an increase in the depletion of AAs by COCs when matured in the presence of urea and EGF.

Next, an individual-IVM culture was used to see the potential differences in the metabolism of AAs based on the developmental competence of COCs. Compared to the group-IVM culture in which the depletion of AAs was the greatest in the presence of high-level urea + EGF, we found that the depletion of AAs was the highest in the high-level urea group in the individual-IVM culture. It has been shown that the culture conditions affect the metabolism of human oocytes^30^. EGF has been shown to increase the blastocyst formation in a single embryo culture system, although EGF did not affect the development of embryos in group culture^31^. The present results showed that the EGF abrogated urea-increased depletion of AAs in the individual-IVM system but synergistically increased depletion of AAs in the group-IVM culture with urea. Mutual interaction between oocyte and CCs may have contributed to different effects of EGF in group and individual IVP systems^32,33^. In other words, the CCs and oocyte maintain the supply of paracrine and autocrine factors, such as EGF, for each other^25^. For example, CCs have been shown to mediate the beneficial effects of EGF on bovine oocyte maturation^2–4^. Therefore, the cooperative relationship between CCs and oocytes is impaired in the individual-IVM system; which may lead to a different response to the presence of EGF in the maturation medium.

In both individual- and group-IVM systems, urea abrogated the EGF-induced the percentage of COCs reached to the cleavage stage. It was found that the percentage of cleavage decreased from 90% found for the EGF group to 54% found for the EGF + urea group, approximately a decrease of 60% in the group-IVM culture (Fig. 4a). However, the percentage of cleavage decreased from 66.7% found for the EGF group to 44.8% found for the EGF + urea group, approximately a decrease of 66% (Table 1). In order to test the interference function of urea on the EGF/EGFR system, two in silico and in vitro experiments (with the use of an EGFR inhibitor, i.e., NAC) were carried out. The in silico data indicated an interference function for urea in binding of EGF to its receptor (EGFR). In addition, an in vitro experiment using NAC showed that urea can act through EGFR. Therefore, these data show that urea can interfere with the positive effects of the EGF/EGFR system in terms of oocyte developmental competence (i.e., the cleavage stage).

Data showed that the developmental competence of COCs matured in the group-IVM culture was improved upon maturation in the presence of the EGF. It was found that the percentage of cleavage increased from 70 to 90% (approximately 28%) in the presence of the EGF in the group-IVM culture. EGF has been reported to mediate oocyte maturation by phosphorylation of mitogen-activated protein kinase (MAPK)^34,35^. In fact, the suppression of EGFR signaling abolishes the acquisition of oocyte developmental competence^34,35^. Previous studies have reported that the MAPK pathway and the EGFR signaling are inhibited by urea and its derivatives^9–11,36^. Oyamada et al. reported that, based on the form of in vitro culture (IVC, i.e., individual-IVC or in group-IVC), the presence of EGF in the maturation medium changes differently the early embryonic development in bovines^33^. These findings again indicate that the nature of the IVM culture (individual system *vs.* group system) and the composition of the culture medium (i.e., the presence of EGF) may have an effect on the competence of the COCs.

As mentioned above, the presence of EGF in the maturation medium improved the subsequent fertilization and cleavage percentage of COCs but did not significantly improve the percentage of COCs reached the blastocyst stage compared to the control group. However, the percentage of blastocyst formation in the EGF group was numerically higher than in the control group (31.03% versus 28.47% for the EGF and the control group, respectively). Similarly, Merriman et al. observed that the fertilization was increased, while the subsequent developmental competence of mouse oocytes matured in the presence of EGF during IVM was impaired^37^. They claimed that the EGF would not be capable of supporting embryonic development^37^. On the other hand, Lonergan et al*.* reported that the presence of EGF during IVM significantly increased the percentage of cleavage and blastocyst formation in bovine COCs isolated from small follicles (2–6 mm)^2^; however, it is known that COCs originating from small follicles (2–4 mm)^35^ are generally non-responsive to the EGF^8,35,38^. In this study, the COCs were recovered from medium sized follicles (7–8 mm) and were expected to react more strongly to the EGF. In addition, in the Lonergan et al*.* study, COCs were matured in a maturation medium containing 10% FCS, which can contain a number of macromolecules, hormones and growth factors^2^. These unknown factors may have an effect on the consequent developmental competence of the COCs, while it was expected that small follicle-derived COCs would not respond to the EGF. In this study, heat-inactivated FBS was used to avoid unknown reactions. It is proposed that differences between the various reports may be due to the fact that conditions during IVM make the resulting embryos more reactive or non-responsive to the culture conditions used for embryo development^37^.

We have previously reported that high urea levels not only increased the percentage of oocyte degeneration, but also decreased the in vitro viability of CCs and oviduct epithelial cells^16,17,39^. In this study, the addition of urea to the maturation medium increased the percentage of oocyte degeneration by day 3 after insemination. Because bovine oocyte expresses aquaporin 3 (AQP3)^40,41^, small solutes, such as water and urea, can readily enter the oocyte. The present data showed that the intracellular content of urea in DOs (cultured for 4 h with 40 mg/dl of urea) was 3.43 mg/dl. In addition, using an in silico method, we found the binding affinity of urea to AQP3 (− 2.79 kcal/mol). This means that urea can enter oocytes using AQP3, providing the opportunity to interact directly with DNA. Moreover, we observed a strong interaction between the urea molecules and the DNA. Likewise, Qiu et al. also found a strong interaction of the hydrogen bond between the urea molecules and DNA bases^42^. Hence, it seems reasonable to assume that urea can bind directly to oocyte genetic material and cause oxidative damage to DNA^43^. This can, in part, explain the higher percentage of COCs degeneration.

In this study, the highest percentage of oocyte degeneration coincided with acute up-regulation of *NANOG* (16.7 folds), *OCT4* (37 folds), *DNMT1* (15.5 folds) and *BAX* (15.7 folds) in blastocysts derived from COCs matured in groups and in the presence of high-level urea and EGF. Previous studies have shown that bovine blastocysts with higher or lower expression of *OCT4* and *NANOG* than normal expression have a poorer potential for further development^44,45^. In addition, the expression of *BCL2* has been shown to be higher in bovine oocytes of good quality^46^. Over-expression of *BAX* is well known to be associated with accelerated apoptotic death^46^. Moreover, the *BCL2 to BAX* ratio can be used to estimate oocytes and embryos for apoptosis or survival^46^. Thus, the 24-h incubation of COCs with high urea levels and the EGF altered the normal pattern of gene expression in the resulting blastocysts. This was also part of the increased degeneration of COCs matured in the presence of urea and EGF.

More specifically, all data (regardless of the experimental treatments) were subjected to a bivariate and PCA analysis to assess the extent and direction of the relationship between AAs and oocyte competence. Indeed, the ANOVA tests cannot cope with complex treatment structures and multidimensional data, i.e., omics data^47^. The Kendall rank correlation identified a significant association between Arg and oocyte developmental competence or degeneration of COCs matured in groups. Interestingly, the bivariate analysis did not detect any significant correlation between the percentage of blastocyst formation and AAs. As regards the relationship between Trp, Val, Arg and the early stages of development (i.e., cleavage stage) but not the blastocyst stage, the advanced stages of reproduction (e.g., blastocyst development, implantation and pregnancy rates) must be recorded upon maturation of COCs. Indeed, embryos developing into the blastocyst stage undergo spontaneous selection during IVC^48^. It indicates that the stage of cleavage does not guarantee the development of embryos after a short-term culture (reflected at a high cleavage proportion) but may reduce the developmental competence of embryos after an extended culture (i.e., 6–7 days)^49^.

In the case of the individual-IVM system, the Pearson correlation identified a significant correlation between Tyr, Thr and the cleaved COCs; this result was different from that observed in the group-IVM system. Next, the PCA analysis was used to evaluate the complex interactions between AAs and the developmental competence of COCs matured individually or in groups^20^. In the case of the group-IVM system, the PCA analysis demonstrated a strong positive association between Arg, Gln, Ile, Trp, Val, and the percentage of COCs reached the 2-PN, cleavage and blastocyst stages (Fig. 6d). However, PCA detected a strong positive association between Ala, Orn, Trp, Glu, Asp, Phe and the proportion of cleaved COCs when COCs matured individually (Fig. 6e). This finding indicated that COCs maturation individually or in groups could change the metabolism of AAs by COCs. Moreover, multivariate analysis appears to outperform bivariate analysis in finding additional factors linked to oocyte developmental competence.

The ROC curve analysis was conducted to identify the best predictor for COCs with a high developmental competence in the group-IVM system and the incidence of cleavage in the individual-IVM system. The AUC analysis showed that, in the case of the group-IVM system, Arg was a biomarker with a good predictive ability to distinguish highly competent oocytes (i.e., high percentage of 2PN, cleavage and blastocyst formation > 80%) from those with low developmental competence (i.e., < 80%). Arginine displayed strong AUC (0.9, on average), sensitivity (87.4%, on average) and specificity (100%, on average) values. Similarly, cluster and network analysis also identified Arg and the percentage of 2PN, cleavage and blastocyst formation in the same cluster. In the network analysis, when an 80% cutoff point was set for network analysis, only Arg was linked to the proportion of COCs reached the 2PN and cleavage stages. Next, to increase the specificity, the cutoff point was increased from 80 to 90% and the results showed that only Arg was related to 2-PN (Fig. 7b). As mentioned above, the increase in Arg depletion coincided with a decrease in the competence of the COCs. Bódis et al. reported an increased level of Arg in follicular fluid, which adversely affected human embryos^50^. They suggested that increased activity of the L-Arg/NO system may have an adverse effect on IVF outcomes^50^. The present findings suggest that Arg depletion (i.e., > − 0.6 pmol/h) may be used as a biomarker during IVM to detect high-competent COCs.

In the case of the individual-IVM culture, the AUC analysis showed that Glu, Ala, Ile, Trp, Met, Orn, Phe, Tyr, and Arg were biomarkers with a good predictive ability to estimate the incidence of COC cleavage. Moreover, the AUC detected Orn and Ala as biomarkers with a good predictive ability to estimate the absence of COC cleavage. These findings were consistent with the results of the PCA, network analysis and the hierarchical dendrogram. The ROC analysis shows that the factors that predict the incidence of cleavage or identify high-competent COCs will also change with changes in the conditions of the IVM.

In conclusion, it can be deduced from these sets of analysis that the conditions of the IVM (i.e., the existence of interactive agents, such as urea and EGF, or the use of individual-IVM and group-IVM cultures) may somehow change the AAs depletion/appearance and developmental competence of the COCs. These findings may provide some insight into the role of urea in reduced fertility of high producing dairy cows fed high-protein diets, but further in vivo experiments are needed to confirm theses in vitro results.

## Methodology

### Animal studies and data statement

All reagents were obtained from Sigma Aldrich (St. Louis, MO, USA) unless otherwise stated. Animal experiments were conducted in accordance with the Guiding Principles for the Care and Use of Research Animals Promulgated by the Isfahan University of Technology, Iran. The protocol and methods were approved by the Committee on the Ethics of Animal Experiments of the Isfahan University of Technology (No. 390132). The datasets are available from the corresponding author upon request.

### In vitro maturation and treatments

From November 2018 to February 2019, bovine ovaries were sourced from the local slaughterhouse and transported to the laboratory within 2 h at about 30 °C using a thermo box containing 0.9% NaCl, 0.1% penicillin, and streptomycin. Antral follicles (7–8 mm in diameter) were isolated from ovaries containing an active corpus luteum and kept at 37 °C in HEPES-TCM-199 (tissue culture medium; Invitrogen, Carlsbad, CA, USA).

In the group-IVM culture (group: 10 COCs per 50-μl droplet), a total of 960 COCs derived from 7–8 mm follicles (from 150 slaughtered cows) were randomized into four experimental groups. All treatments were repeated six times (6 independent experiments). At each replication, COCs from different cows were pooled and randomly assigned into treatment groups. On average, a total of 24 wells as the experimental units (24 droplets with 10 COCs per each) were assigned to each experimental treatment (Supplementary Fig. 1, Phase 1). Only COCs with at least five cell layers were selected under a stereomicroscope (Olympus, Tokyo, Japan) and transferred to HEPES-TCM-199 supplemented by 50 mg/ml of kanamycin and 50 mg/ml of heparin. Intact COCs were then washed twice in HEPES-TCM199 and randomly placed in groups of 10 in 50-µl droplet of maturation medium containing bicarbonate-buffered TCM199 supplemented by 10% (w/v) of heat-inactivated fetal bovine serum (FBS, BioWhittaker, Walkersville, MD, U.S.A.) and 20 IU/ml follicle-stimulating hormone (FSH) in 35 mm cell culture dishes (Falcon brand, BD Biosciences, San Jose, CA, U.S.A.) for 24 h at 38.5 °C under 5.5% CO_2_, 20% O_2_, balanced N_2,_ and maximum humidity under mineral oil. The experimental treatments were as follows: (1) maturation medium without urea and EGF (control group, n = 250 COCs); (2) maturation medium with 10 ng/ml EGF (E group, n = 230 COCs); (3) maturation medium with 10 ng/ml EGF and 20 mg/dl urea (EU20 group, n = 240 COCs); and (4) maturation medium with 10 ng/ml EGF and 40 mg/dl urea (EU40 group, n = 240 COCs). Following IVM, the 24-h spent maturation medium (24 samples from 24 wells per treatment) was kept at -70 until HPLC analysis.

In the individual-IVM culture (individual: 1 COC per 10-μl droplet), a total of 366 COCs were randomized to four experimental groups. Intact COCs were washed twice in HEPES-TCM199 and placed randomly individually in 10-µl droplet of the maturation medium overlaid with paraffin liquid. The individual-IVM culture was conducted under the same conditions as those described above for the group-IVM culture. The experimental treatments used for individual-IVM culture were as follows: (1) maturation medium without urea and EGF (control group, n = 90 COCs); (2) maturation medium with 10 ng/ml EGF (E group, n = 87 COCs); (3) maturation medium with 40 mg/dl urea (n = 93 COCs); and (4) maturation medium with 10 ng/ml EGF and 40 mg/dl urea (EU group, n = 96 COCs). Following IVM, the 24-h spent maturation medium (12 samples per treatment) was randomly selected from each treatment on the basis of the cleavage status (cleaved and non-cleaved COCs) and kept at − 70 until HPLC analysis.

NAC was used to test the possible effects of urea on AAs via EGFR system. NAC has been shown to inhibit EGFR activation^29^. Urea signaling has been shown to be sensitive to NAC^29^. Using the group-IVM culture (group: 10 COCs per 50-μl droplet), a total of 360 COCs were randomized into three experimental groups as follows: (1) maturation medium with no supplementation (control group, n = 60 COCs); (2) maturation medium with 40 mg/dl urea (n = 60 COCs); and (3) maturation medium with 40 mg/dl urea + 1 mM NAC (n = 60 COCs). The IVM was conducted under the same conditions as those described above for the group-IVM culture. After IVM, the 24-h spent maturation medium (6 samples per treatment) was randomly selected from each treatment and kept at -70 until HPLC analysis.

In the present study, COCs, but not DOs, MII-oocytes or in vitro-synchronized oocytes, were used for the assessment of AAs depletion/appearance. In addition, COCs were specifically drawn from 7–8 mm follicles to maintain the same maturation stage as possible and to minimize variability in the amount of cumulus layers. Since there is a cross-talk between oocyte and its accompanying CCs, which plays an important role in metabolism, in AAs uptake by oocytes, and in the subsequent developmental competence of oocytes^51^, COCs were not denuded in this study.

Based on the concentrations of urea in the blood and follicular fluid of dairy cows fed a low- or high-protein diet, 20 mg/dl urea (equivalent to 9.3 mg/dl BUN) and 40 mg/dl urea (equivalent to 18.7 mg/dl BUN) were used in this study^13,52,53^.Therefore, these levels of urea simulated the BUN values found in healthy dairy cows fed low- or high- protein diets, respectively^53,54^.In fact, the concentration of urea in follicular fluid is similar to that in plasma as it quickly spreads to body fluids, such as follicular fluid^13^. In this study, the EGF concentration (10 ng/ml) was based on the minimum effective dose reported in previous reports^2^. Follicular concentrations of EGF have been reported to vary from 2 to 15 ng/ml^2^.

### Amino acid and urea determination

AAs were measured using HPLC, before HPLC analysis seven samples (seven out of 24 samples of 24-h IVM-wells per treatment) that were contaminated with oil were discarded; ultimately 17 droplets or wells (i.e., experimental units) per treatment were selected for the AAs analysis by HPLC (Supplementary Fig. 1, Phase 1). The determination of AAs in the 24-h spent media (n = 17) and fresh maturation media (n = 6) was conducted using the HPLC method (HyperClone ODS C 18, 250 mm × 4.6 mm, 5 μm, Agilent 1100, Agilent Technologies, Waldbronn, Germany) based on our previous work^17^. HPLC was used at a flow rate of 1.2 ml/min with fluorescence detection after pre-column derivatization (with o-phthaldialdehyde, OPA). The fluorescent signal was 450 nm when it was excited at 348 nm. As the reference (blank), maturation medium without COCs, EGF and urea supplementation were cultured under the same conditions as the test samples. After 24-h incubation, all blanks and test media were immediately kept at -70 °C and analyzed using the same protocol.

For the determination of AA concentrations, the peak areas of the samples and the standard AA mixture were compared. Using peak signals from internal standards (25 mM sarcosine and 25 mM norvaline both in 0.1 M HCl as internal standards), all peak signals were normalized. O-phthaldialdehyde in the presence of the thiol component (OPA method) used in this study has been shown to have a low response to cystine and none to proline^55^. The assay of asparagine was unlikely due to conversion to aspartic acid during the preparation of the sample^56^.

Amino acids were classified as (1) EAAs, including leucine (Leu), lysine (Lys), phenylalanine (Phe), isoleucine (Ile), valine (Val), methionine (Met), tryptophan (Trp), threonine (Thr), and histidine (His); (2) semi-EAAs, including serine (Ser), arginine (Arg), tyrosine (Tyr), glycine (Gly), and glutamine (Gln), and (3) non-EAAs, including aspartate (Asp), alanine (Ala), citrulline (Cit), ornithine (Orn), and glutamate (Glu), according to previous works^17,56,57^. However some AAs, such as tyrosine and arginine, are semi-essential for cows, it should be remembered that certain types of cells (e.g., CCs or oocytes) may not be capable of producing such AAs, and therefore these AAs are essential^58^.

The accuracy of the AA assessment was checked by calculating the mean recovery of the biological medium with a certain AAs volume close to that of the AAs in the maturation medium. Accuracy was defined as the agreement between the measured value and the actual AAs value in the reference media. The average recovery for all AAs observed was 105.8%. The accuracy ranged from 91.1% to 109.1%, excluding aspartic acid (116.4%). Therefore, the limit for all AAs was set at between 90 and 117%. In order to test the precision of the method, the repeatability was calculated by injecting the same samples of fresh maturation medium (four times) in a row and calculating the relative standard deviation (*RSD*) for each AA. The *RSD* values obtained were within an acceptable range of 1.15 to 3.59%. The average statistical power of this experiment was adequate at 88.9 ± 10.0%, ranging from 68.0 to 100%.

In order to determine intracellular levels of urea, DOs in groups of 25 were cultured in the culture medium (50 µl droplet) for 4 h with 40 mg/dl urea. The harvested DOs were washed and centrifuged at 300* g* for 5 min and re-suspended in the culture medium (1 ml). The resultant mixture was mechanically lysed with ultrasonic homogenization for 1 min, and then urea was measured on the Hitachi 917 auto-analyzer (Roche Diagnostics, Mannheim, Germany) using an enzymatic method.

### Calculation of amino acid depletion/appearance

Differences between the concentration of AA in the test medium (after 24 h of incubation) and that of fresh medium were used to calculate the depletion or appearance of each AA^18,19^. The concentration of AAs in fresh maturation medium was based on concentrations present in the tissue culture medium (TCM)-199 and previous studies^59^. In recent studies, the depletion/appearance of AAs in bovine and human MII oocytes was measured during the final 6 h of IVM with a decreased concentration of AA (one-sixth MEM and five-sixth Earle’s Balanced Salt Solution)^18,19^. In previous studies by Hemmings et al., IVM was divided into two different phases, including phase I in which the maturation media had a normal AA concentration and phase II in which the AA concentration was reduced at the final 6 h of IVM^18,19^. They measured the AAs concentration of spent media of the last 6 h of IVM. On the other hand, we did not transfer COCs to a fresh maturation medium with a reduced concentration of AA (e.g., for the last 6 h of IVM) and measured the concentration of AA in a 24-h spent medium.

It has been shown that, in the mammalian cell culture model, sudden nutrient reductions, such as AAs, can quickly stimulate autophagy within minutes^60^. In addition, it has been confirmed that MII oocytes have completed the final maturation processes, with reduced synthetic activity and a quiet stage^22^. As a consequence, we used the 24-h spent medium for AA determination to cover all phases of COCs metabolism. In addition, in order to reduce possible adverse reactions, such as autophagy^60^, we did not transfer COCs to the reduced AA concentration media during IVM (e.g., for the last 6 h of IVM).

Data were calculated in pmol/h and adjusted on the basis of the total number of COCs (n = 10) within each droplet, according to previous reports^18,19^. In addition, the concentrations of AAs in each 24-h spent medium were normalized to the number of CCs derived from 10 COCs inseminated in each droplet. It was performed to reduce the effect of variability (in the amount of CCs attached to COCs) on the depletion/appearance of AAs. To estimate the number of CCs per each droplet (consisting 10 COCs), the number of fertilizing sperm cells (2 × 10^6^/ml) was subtracted from the total cell numbers (including sperm and CCs) counted 8 h after IVF. Cumulus cells were completely removed from the oocytes through gentle vortex agitation. Approximately (2.6 ± 0.2) × 10^5^ cells, varying from (1.9 ± 0.2) × 10^5^ to (2.7 ± 0.8) × 10^5^, were counted per each droplet. The total number of cells (dead and live) was evaluated using Trypan blue staining.

### In vitro fertilization and embryo culture

After IVM (using a group culture), COCs from two droplets of the same treatment were pooled in order to achieve a well (50-μl droplet) of 18 to 20 COCs. Therefore, COCs were allocated to 12 wells (50-μl droplet) and to four groups based on their own IVM treatments (Supplementary Fig. 1, Phase 2). Thus, the IVF and IVC media were not treated with any agents (e.g., EGF and urea). Next, COCs matured under different experimental treatments during IVM were inseminated with sperm cells at a final concentration of 2 × 10^6^/ml in the IVF medium (50-μl droplets) and categorized as follows: (1) fertilizing sperm + COCs matured in the absence of urea and EGF (the control group); (2) fertilizing sperm + COCs matured in the presence of 10 ng/ml EGF; (3) fertilizing sperm + COCs matured in the presence of 10 ng/ml EGF and 20 mg/dl urea; and (4) fertilizing sperm + COCs matured in the presence of 10 ng/ml EGF and 40 mg/dl urea.

In the case of individual-IVM culture, following IVM, COCs were individually transferred to IVF medium (20-μl droplet), inseminated with sperm cells at a final concentration of 2 × 10^6^/ml and categorized as follows: (1) fertilizing sperm + COCs matured without urea and EGF (the control group); (2) fertilizing sperm + COCs matured in the presence of 10 ng/ml EGF; (3) fertilizing sperm + COCs matured in the presence of 40 mg/dl urea; and (4) fertilizing sperm + COCs matured in the presence of 10 ng/ml EGF and 40 mg/dl.

The insemination of COCs was performed in the IVF medium, supplemented by BSA in 35 mm cell culture dishes (Falcon brand, BD Biosciences, San Jose, CA, USA) at 38.5 °C under 5% CO_2_ in air. According to a previous study^61^, sperm cells were capacitated in a 96-well untreated polystyrene microplate (Corning Incorporated, New York, U.S.A.) using a modified Tyrod’s albumin, lactate, and pyruvate medium (Sp-TALP). In brief, after 20 min of swim-down, sperm cells (5 × 10^6^ sperm/ml) were suspended in Sp-TALP supplemented with 10 mg/ml heparin, and incubated for 18 h.

Zygotes were recovered 8 h after insemination and CCs were manually separated by repeated pipetting in HEPES-buffered MEM (BioWhittaker, Walkersville, MD, U.S.A.) containing 80 IU/ml of bovine hyaluronidase using narrow-bore glass pipettes. The resulting embryos were transferred to 35 mm cell culture dishes containing synthetic oviduct fluid medium (50-μl droplet), supplemented by 30 µl/ml of EAAs solution (50 × ; 11130-051; Gibco), 10 µl/ml of non-EAAs solution (100 × ; 11140; Gibco), and 4 mg/ml BSA in an incubator at 38.5 °C, 5% O_2_, 6% CO_2_, and 90% N_2_. The proportion of COCs which did not reach the 2-PN stage was recorded using a stereomicroscope (Olympus, Tokyo, Japan) at 18 h post-insemination. The percentage of COCs reached the cleavage stage (2–16 cell stage, day 3 post-insemination) or blastocyst stage (day 7 post-insemination) was reported. All developmental parameters, including 2PN, cleavage, and blastocyst percentage, were calculated in relation to the initial number of COCs cultured in the maturation medium. Eventually, a total of nine pools were derived from 12 IVC droplets per treatment (Supplementary Fig. 1, Phase 3). Each pool consisted of 7 to 8 blastocysts, which produced from COCs that matured under different treatments (i.e., urea and EGF) and kept at − 70 until qRT-PCR. These embryos had been cultured in IVC media based on initial experimental treatment used for COCs and had not been treated with any agents (e.g., EGF and urea).

### RNA extraction, reverse transcription, quantitative analysis of transcripts using qRT-PCR

RNA extraction and reverse transcription were conducted as reported earlier^16^. In short, total RNA was extracted from day-7 blastocysts using RNeasy Micro kit (Qiagen, Mississauga, Ontario, Canada) and then treated with DNase I (Ambion, Streetsville, Ontario, Canada) according to the manufacturer’s manual. To determine the quality and quantity of RNA, the WPA Biowave spectrophotometer (Cambridge, United Kingdom) was employed. Reverse transcription was done for 10 min at 25 °C, for 1 h at 42 °C, and for 10 min at 70 °C. Using 1 µl of cDNA (50 ng), 5 µl of the SYBR Green/0.2 µl of ROX qPCR Master Mix (2X, Fermentas, Germany) and 1 µl of forward and reverse primers (5 pM) adjusted to a total volume of 10 ml using nuclease-free water, qRT-PCR was carried out. The reference gene was *ACTB*. Each replicate of qRT-PCR was repeated three times in order to minimize the technical errors. The primer sequences that were used for qRT-PCR were: 5′-CCTTCTTTGAGTTCGGAG-3′, forward, and 5′-CCTTCAGAGACAGCCAG-3′, reverse, for *BCL2* (AC_000181.1, product size (bp) 121, Tm: 60 °C); 5′-AGCGAGTGTTCTGAAGCG-3′, forward, and 5′-CCCAGTTGAAGTTGCCGT-3′, reverse, for *BAX* (AC_000175.1, 182 bp, , Tm: 61 °C); 5′-GGTGTTTGGTGAACTCTC-3′, forward, and 5′-CATTGATTGTTCCAAGGCT-3′, reverse, for *NANOG* (AC_000162.1, 98 bp, Tm: 53 °C); 5′-GGAAAGGTGTTCAGCCA-3′, forward, and 5′-TTCTCGTTGTTGTCAGC-3′, reverse, for *OCT4* (NC_000006.12, 131 bp, Tm: 62 °C); 5′-GAAGCAGAATAAGAATCGG-3′, forward, and 5′-TTTGAAGAGTCGTCTGGAA-3′, reverse, for *DNMT1* (AC_000164.1, 125 bp, Tm: 53 °C); 5′-CCATCGGCAATGAGCGGT-3′, forward, and 5′- CGTGTTGGCGTAGAGGTC-3′, reverse, for *ACTB* (AC_000182.1, 146 bp, Tm: 60 °C).

Despite normal morphology, the condition of IVM has been shown to have a negative effect on the level of gene expression in the human preimplanted embryos^62^. Therefore, we assumed that different IVM conditions in this study could affect the gene expression in the resulting blastocysts. Warzych et al. reported that supplementation of the maturation medium with a macromolecule (PVP40) was associated with a higher apoptotic index in bovine blastocysts^63^. Moreover, because urea as a toxin was used during IVM in this study, we decided to choose *BCL2* and *BAX* transcripts to determine whether the IVM environment could affect the major apoptosis regulators in the resulting blastocysts. In addition, EGFR inhibition increases the rate of apoptosis and changes the expression of specific BCL2 family members in favor of apoptosis^64^. EGFR has been shown to be clearly associated with the transcription factors OCT4 and NANOG, which are essential for determining the pluripotent status of embryonic stem cells^65^. EGFR inhibition has been reported to reduce the expression of *OCT4* and *NANOG,* while the activation of EGFR acutely increases the activity of DNA methyltransferase (DNMT)^65,66^. Hence, these transcription factors were selected in this study to be evaluated in the resulting blastocysts.

### Statistics

The Anderson–Darling test (EasyFit software, version 5.6, MathWave Technologies, Spokane, WA, USA) showed that the data from the group-IVM culture was normally distributed in each experimental treatment, but the data was non-normally distributed regardless of the experimental treatments. In the case of individual-IVM culture, data was normally distributed in each experimental treatment or regardless of experimental treatments. The medians of depletion/appearance of AAs and developmental competence of COCs were analyzed using the Kruskal–Wallis test followed by the Dunn’s multiple comparison test. In the case of gene expression and use of NAC, the one-way ANOVA followed by Tukey’s multiple comparisons test was used to evaluate the results. Differences at *p* < 0.05 were considered statistically significant. Orthogonal polynomial contrasts were used to determine the linear or quadratic effect of urea using SAS software (SAS Institute Inc., Cary, NC). For ANOVA analysis study, we also calculated *q-*value *vs*. *p-*value to address multiple testing problems. Multiple testing problems usually exist in metabolomic-based studies^67^. Indeed, a *p*-value of 0.05 is a false positive rate when one test is done, but with a large number of tests on the data, one needs to compensate with false positives. There are various approaches to correct for multiple testing. The use of false discovery rates (i.e., *q*-values) is most common. The *q*-value represents the false discovery rate; the lower *q*-value (*q* < 0.10) implies high confidence in the test^67^. In this analysis, we observed that the false discovery rate of various comparisons with significant *p* values was small (*q < *0.08, Supplementary Fig. 6 and Supplementary Fig. 7). According to *p*-value *vs .q*-value plot, in these results, we found that *q*-value 0.05 corresponded *p*-value of about 0.03 (Supplementary Fig. 7a). For this analysis, threshold comparisons of *q*-values of less than 0.05 yield 113 significant differences between comparisons (Supplementary Fig. 7b,c). It indicates that only 6 of the 113 comparisons listed significant are likely to be false positives.

The bivariate analysis was performed using Kendall’s tau or Pearson correlation (for the data derived from the group- and individual-IVM systems, respectively) to determine the associations between each parameter relevant to the developmental competence and AAs depletion/appearance. Next, multivariate methods, such as the Hierarchical Clustering Analysis (HCA) and the Principal Component Analysis (PCA), were used to consider the effects of inter-correlated variables (i.e., AAs) simultaneously on IVF outcomes. Bonferroni correction was used to test the significance of the correlations in the correlation matrix or in the t-test analysis.

Because, the data derived from the group-IVM culture were not normally distributed, these non-parametric data were transformed using the logarithm function. These log-transformed data were then applied to HCA and PCA and analyzed using the Kendall distance metric. However, Pearson metric was used in the case of normal data derived from the individual-IVM culture.

Using the “Heatmapper” web tool (https://www2.heatmapper.ca/expression/), an HCA analysis with Kendall or Pearson distance metrics (for data derived from group- and individual-IVM systems, respectively) and a complete linkage method was used to produce clustering patterns of AAs and developmental competence. The PCA analysis was used to consider the effects of all AAs on oocyte competence at the same time, irrespective of experimental treatments, to reduce the multiple data dimensions to two dimensions and to produce biplots.

Network analysis was performed to identify central nodes (i.e., AAs). Rho and Pearson similarity indexes were used for data derived from group- and individual-IVM systems, respectively. The Fruchterman-Reingold algorithm was used as a force-directed layout algorithm. To this aim, the data was mapped to the amino acid-developmental competence interaction network. For the Kendall rank correlation, ANOVA, PCA, network mapping, and log transformation, the PAST program (accessible at: https://folk.uio.no/ohammer/past) was used.

Using the easyROC web-tool (https://www.biosoft.hacettepe.edu.tr/easyROC/), a Receiver Operating Characteristic (ROC) curve analysis was performed to identify the best predictive factors for the detection of COCs with high developmental competence. The optimal cutoff for high IVF outcome prediction was determined by maximizing the Youden index. In order to deal with potential biases in the results produced by assuming that any relationship between AAs and high oocyte competence could be linear, AAs were divided into two separate groups based on IVF outcomes derived from the group-IVM system , i.e., high percentage of 2PN (> 80%) *vs.* absence of high 2PN percentage (< 80%), high percentage of cleavage (> 80%) *vs.* absence of high percentage of cleavage (< 80%), high percentage of blastocyst formation (> 30%) *vs.* absence of high percentage of blastocyst formation (< 80%), and low percentage of oocyte degeneration (< 15%) *vs.* absence of low percentage of oocyte degeneration (> 15%). Also, AAs were divided into two separate groups based on the incidence of cleavage (incidence of cleavage *vs.* absence of cleavage) in the case of an individual-IVM system. Finally, the area under the curve (AUC) was determined by the Mann–Whitney statistic (a non-parametric unbiased likelihood estimator) to detect COCs with a high percentage of 2PN, cleavage and blastocyst formation, as well as low percentage of oocyte degeneration. In the case of an individual-IVM culture with normal data, the AUC was determined by the Asymptotic statistic (a parametric unbiased likelihood estimator) for the detection of the competent COCs.

### In silico molecular docking

The initial structure of urea was constructed by GaussView 5.0, which is a graphical software widely used with Gaussian^68^. The optimized structure of urea was obtained using GAMESS-US package^69^ at the B3LYP/6–31 + G (d) level through density functional theory (DFT).

### Molecular docking to predict urea interactions with DNA, EGF, EGFR, and AQP3

Molecular docking procedure is widely recognized as one of the important useful tools for computational biology and chemistry^70,71^. This approach can be used to study the binding free energy and ligand binding activity to receptors^72^. Using AutoDock 4.2 tools, the optimized urea was docked with the crystal structure of DNA, EGF, EGFR and AQP3 to predict the binding energy of urea with these molecules^73^. Urea (without any rotatable bond) and other molecules were respectively regarded as ligand and receptors. Molecular dockings were performed using a Lamarkian Genetic Algorithm to predict binding free energy and the direction of urea in its interaction with DNA, EGF, EGFR and AQP3. The grid sizes of "82 × 84 × 126", "82 × 84 × 126", "126 × 126 × 126", and "126 × 40 × 40" were used for the urea-DNA, urea-EGF, urea-EGFR, and urea-AQP3 complexes, respectively. Also, grid spacing of 0.39, 0.45, 1, and 0.375 Å was used for the urea-DNA, urea-EGF, urea-EGFR, and urea-AQP3 complexes, respectively. The crystal form of the extracellular domain of EGFR (PDB ID: 3NJP), EGF (1jl9), and DNA (1 BNA) was taken from the Protein Data Bank at a resolution of 3.30, 3.90, 1.0 Å, respectively. Computer modeling on AQP3 (ID: Q08DE6) was done by using AA sequences from the Universal Protein Resource (UniProt) database (https://www.uniprot.org), accompanied by 3-D structure predictions in the I-TASSER repository (https://zhanglab.ccmb.med.umich.edu/I-TASSER). The blind docking was completed to discover the suitable binding sites for the urea molecule’s interaction with the receptors. The blind docking scans the entire receptor surface and calculates the positions of the highest binding affinity. The focus docking was performed after the blind docking had been completed. The total number of docking runs for all urea-DNA, urea-EGF, urea-EGFR, and urea-AQP3 docking procedures was set at 100.

In order to study the urea binding behavior to different receptors, the docking procedure was performed for the urea-DNA, urea-EGF, urea-EGFR, and urea-AQP3 complexes at 20, 12, 12, and 12 sequential times, respectively. To this aim, the previous urea-receptor complex was used as a receptor for the next molecular docking (under the same conditions). For example, the urea-EGFR complex with *n* urea molecules was used as the initial template for obtaining the urea/EGFR complex with n + 1 urea molecules. It should be noted that when the next urea molecule was added to the complex, the positions of the urea molecules in the urea/receptor complex obtained from the preceding docking were rigid.

Next, to investigate how EGF’s binding energy with EGFR changes in the presence of urea, the molecular docking was performed as follows with four phases: phase I: EGF/EGFR; phase II: (EGF-urea complex)/EGFR; phase III: EGF/(EGFR-urea complex); and phase IV: (urea-EGF complex)/(EGFR-urea complex). The EGF- urea and EGFR-urea complexes obtained from the preceding step were used as receptor in these phases. Using Lamarkian Genetic Algorithm, molecular dockings were performed to predict binding free energy of these complexes. The grid sizes of "126 × 104 × 94", "76 × 126 × 92", "105 × 126 × 82" and "82 × 96 × 68" were used for phases I, II, III and IV, respectively. Also, grid spacing of 0.669, 0.686, 0.664 and 1 Å was used for phases I, II, III and IV, respectively. LIGPLOT software was used to identify van der Waals interactions between urea and residues^74^. LIGPLOT is a bioinformatics computer program that produces 2-D ligand-receptor interaction representation^74^.

## Supplementary information

Supplementary Information.
