# Supplementary material for: A multilevel analysis identifies the different relationships between amino acids and the competence of oocytes matured individually or in groups
Source: Sci Rep. 2020 Sep 30;10:16082. doi: 10.1038/s41598-020-73225-7 (PMC7528030; doi:10.1038/s41598-020-73225-7)
Supplement: Supplementary file 1 — Supplementary Information. [file 41598_2020_73225_MOESM1_ESM.pdf]

Supplementary Information

A multilevel analysis identifies the different relationships between amino acids and the competence of oocytes matured individually or in groups

Rasoul Kowsar[1,2,*], Alireza Mansouri[2], Nima Sadeghi[3], Mohammad Heidaran Ali Abadi,[1] Seyed Mehdi Ghorieshi[4], Khaled Sadeghi[1], and Akio Miyamoto[2]

[1] Department of Animal Sciences, College of Agriculture, Isfahan University of Technology, Isfahan, 84156–83111, Iran.

[2] Graduate School of Animal and Food Hygiene, Obihiro University of Agriculture and Veterinary Medicine, Obihiro, Hokkaido, 080-8555, Japan.

[3] FKA, Animal Husbandry and Agriculture Co, Isfahan, Iran.

[4] Department of Animal Sciences, College of Agriculture, Shiraz University, Shiraz, Iran.

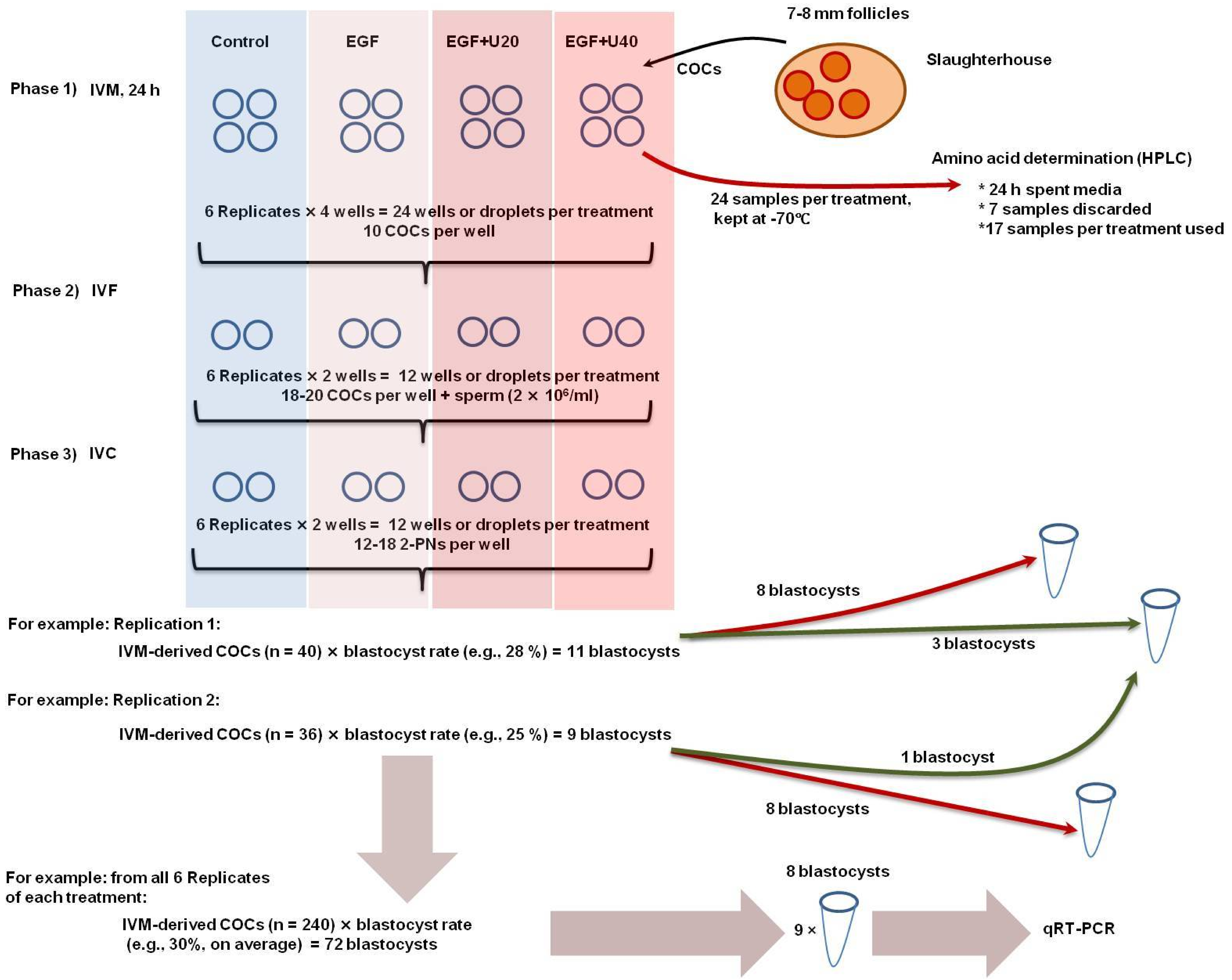

Control
EGF
EGF+U20
EGF+U40
7-8 mm follicles
COCs
Slaughterhouse
Phase 1) IVM, 24 h
Amino acid determination (HPLC)
* 24 h spent media
* 7 samples discarded
*17 samples per treatment used
6 Replicates × 4 wells = 24 wells or droplets per treatment
10 COCs per well
24 samples per treatment,
kept at -70℃
Phase 2) IVF
6 Replicates × 2 wells = 12 wells or droplets per treatment
18-20 COCs per well + sperm (2 × 10⁶/ml)
Phase 3) IVC
6 Replicates × 2 wells = 12 wells or droplets per treatment
12-18 2-PNs per well
8 blastocysts
For example: Replication 1:
IVM-derived COCs (n = 40) × blastocyst rate (e.g., 28 %) = 11 blastocysts
3 blastocysts
For example: Replication 2:
IVM-derived COCs (n = 36) × blastocyst rate (e.g., 25 %) = 9 blastocysts
1 blastocyst
8 blastocysts
8 blastocysts
For example: from all 6 Replicates
of each treatment:
IVM-derived COCs (n = 240) × blastocyst rate
(e.g., 30%, on average) = 72 blastocysts
9 ×
qRT-PCR

Supplementary Fig. 1. Schematic representation of the experimental model. **Phase 1)** A total of 960 COCs derived from 7-8 mm follicles (from 150 cows) were randomized to treatment groups at six time points (6 independent experiments); on average, a total of 24 wells (i.e., 24 droplets with 10 COCs) were assigned to each experimental treatment. Following IVM, the 24-h spent maturation medium (24 samples from 24 wells per treatment) was kept at -70 until HPLC analysis. Before the HPLC analysis, seven samples (seven out of 24 samples of 24-h IVM-wells per treatment) that were contaminated with oil were discarded; ultimately 17 samples per treatment were selected for the AAs analysis by HPLC; **Phase 2)** Next, COCs matured under different experimental treatments during IVM were inseminated with sperm cells at a final concentration of 2×10^6/ml in the IVF medium (50-µl droplets); and **Phase 3)** Upon IVM, COCs derived from two droplets of the same treatment were pooled in order to achieve a well (50-µl droplet) of 18 to 20 COCs. Therefore, COCs were allocated to 12 wells (50-µl droplets) and four groups based on their own IVM treatments; thus, IVF and the in vitro culture (IVC) medium were not treated with any agents (e.g., EGF and urea). Eventually, for qRT-PCR, blastocysts (pool of seven to eight blastocysts (day 7) per treatment per replication, a total of nine replicates) derived from COCs matured under the abovementioned experimental treatments (i.e., EGF and urea) were kept at -70 until analysis. U20: 20 mg/dl urea; U40: 40 mg/dl urea.

Supplementary Fig. 2. The bivariate analysis using Kendall's tau correlation was performed to evaluate the associations between each parameter related to the developmental competence and AAs depletion/appearance by cleaved COCs matured in groups.

-1.00	-0.80	-0.60	-0.40	-0.20	0.00	0.20	0.40	0.60	0.80	1.00

	Asp	Glu	Ser	Gln	His	Gly	Thr	Cit	Arg	Ala	Tyr	Val	Met	Trp	Phe	Ilu	Orn	Leu	Lys	2PN rate	Cleavage rate	Blastocyst rate
Glu	0.47																					
Ser	-0.21	-0.38																				
Gln	-0.12	0.11	0.48																			
His	-0.09	0.38	-0.09	0.18																		
Gly	0.27	0.75	-0.39	0.12	0.21																	
Thr	0.15	0.11	0.39	0.30	-0.15	0.09																
Cit	0.16	0.68	-0.39	0.07	0.30	0.63	0.13															
Arg	0.03	-0.08	0.33	0.36	-0.39	0.09	0.27	-0.20														
Ala	0.21	0.63	-0.03	0.36	0.39	0.64	0.45	0.63	0.09													
Tyr	0.35	0.77	-0.17	0.29	0.53	0.63	0.32	0.68	-0.05	0.87												
Val	-0.27	-0.26	0.52	0.24	0.09	-0.09	0.58	-0.10	0.21	0.09	-0.05											
Met	-0.27	0.20	-0.21	0.00	-0.21	0.39	0.33	0.49	-0.03	0.33	0.20	0.15										
Trp	-0.35	-0.06	0.29	0.35	0.17	0.14	0.50	0.15	0.26	0.32	0.18	0.78	0.38									
Phe	-0.11	0.25	0.14	0.23	0.02	0.44	0.66	0.41	0.23	0.63	0.49	0.47	0.66	0.65								
Ilu	0.09	0.11	0.27	0.30	0.03	0.09	0.82	0.13	0.15	0.45	0.32	0.39	0.21	0.38	0.50							
Orn	0.03	0.44	0.09	0.48	0.39	0.45	0.45	0.56	0.03	0.76	0.69	0.27	0.33	0.50	0.63	0.45						
Leu	0.12	0.32	0.30	0.45	0.06	0.30	0.79	0.36	0.24	0.67	0.53	0.42	0.36	0.47	0.66	0.79	0.67					
Lys	0.18	0.56	-0.12	0.27	0.48	0.55	0.36	0.53	0.00	0.85	0.78	0.00	0.18	0.17	0.47	0.55	0.67	0.58				
2PN rate	0.18	0.08	0.36	0.39	-0.12	0.06	0.42	-0.07	0.73	0.30	0.17	0.24	-0.06	0.29	0.20	0.30	0.18	0.39	0.21			
Cleavage rate	0.09	0.05	0.03	0.24	-0.03	0.15	0.27	-0.10	0.58	0.21	0.08	0.21	-0.09	0.26	0.17	0.33	0.03	0.24	0.30	0.55		
Blastocyst rate	0.12	0.14	0.12	0.22	-0.15	0.09	0.37	0.13	0.40	0.25	0.20	0.12	0.00	0.17	0.20	0.31	0.12	0.34	0.28	0.49	0.34	
Degeneration rate	-0.22	-0.14	0.00	-0.22	0.18	-0.31	-0.34	-0.10	-0.62	-0.34	-0.20	-0.25	-0.06	-0.29	-0.29	-0.22	-0.09	-0.31	-0.25	-0.65	-0.62	-0.53

	Asp	Glu	His	Ser	Gln	Arg	Cit	Gly	Thr	Ala	Tyr	Met	Val	Phe	Ile	Leu	Orn	Lys	Trp
Glu	0.15																		
His	-0.08	0.54																	
Ser	0.08	-0.09	0.61																
Gln	0.22	0.52	0.94	0.66															
Arg	0.37	-0.34	0.37	0.89	0.53														
Cit	-0.42	0.21	0.50	0.64	0.37	0.29													
Gly	0.31	0.12	0.76	0.90	0.86	0.86	0.44												
Thr	0.75	0.22	0.48	0.63	0.72	0.74	0.12	0.84											
Ala	0.36	0.93	0.39	-0.25	0.46	-0.35	-0.07	0.05	0.30										
Tyr	0.90	0.24	-0.03	0.23	0.24	0.40	-0.05	0.38	0.79	0.35									
Met	-0.24	0.57	0.95	0.61	0.87	0.29	0.64	0.66	0.33	0.37	-0.13								
Val	0.02	0.21	0.74	0.94	0.75	0.72	0.78	0.87	0.60	-0.01	0.24	0.77							
Phe	-0.02	0.71	0.93	0.53	0.89	0.25	0.62	0.69	0.51	0.56	0.14	0.92	0.73						
Ile	-0.07	0.37	0.95	0.79	0.92	0.57	0.66	0.87	0.57	0.23	0.06	0.92	0.87	0.91					
Leu	-0.06	0.01	0.72	0.92	0.71	0.76	0.73	0.90	0.59	-0.16	0.14	0.68	0.92	0.68	0.88				
Orn	0.19	0.93	0.35	-0.31	0.36	-0.47	-0.08	-0.10	0.08	0.94	0.16	0.40	-0.05	0.48	0.14	-0.29			
Lys	0.50	-0.28	0.34	0.69	0.54	0.88	-0.10	0.75	0.71	-0.20	0.34	0.22	0.49	0.13	0.43	0.49	-0.27		
Trp	0.48	0.83	0.32	-0.32	0.40	-0.34	-0.26	0.06	0.35	0.92	0.42	0.22	-0.10	0.45	0.11	-0.19	0.84	-0.15	
Cleaved	0.43	0.57	0.33	0.33	0.39	0.19	0.45	0.43	0.58	0.44	0.71	0.31	0.53	0.55	0.36	0.41	0.32	-0.02	0.49

-1.00 -0.80 -0.60 -0.40 -0.20 0.00 0.20 0.40 0.60 0.80 1.00

Supplementary Fig. 3. The bivariate analysis using Pearson correlation was performed to evaluate the associations between the rate of cleaved COCs and AAs depletion/appearance by COCs matured individually.

Supplementary Fig. 4. The bivariate analysis using Pearson correlation was performed to evaluate the associations between the rate of non-cleaved COCs and AAs depletion/appearance by COCs matured individually.

-1.00	-0.80	-0.60	-0.40	-0.20	0.00	0.20	0.40	0.60	0.80	1.00

	Asp	Glu	His	Ser	Gln	Arg	Cit	Gly	Thr	Ala	Tyr	Met	Val	Phe	Ile	Leu	Orn	Lys	Trp
Glu	0.96																		
His	0.94	0.95																	
Ser	0.87	0.87	0.90																
Gln	0.97	0.96	0.99	0.91															
Arg	0.88	0.91	0.90	0.98	0.92														
Cit	0.90	0.80	0.82	0.90	0.87	0.89													
Gly	0.92	0.91	0.94	0.99	0.95	0.98	0.92												
Thr	0.78	0.79	0.92	0.89	0.86	0.84	0.73	0.90											
Ala	0.80	0.79	0.89	0.65	0.85	0.63	0.56	0.71	0.79										
Tyr	0.94	0.90	0.80	0.81	0.85	0.85	0.90	0.84	0.62	0.56									
Met	0.63	0.63	0.82	0.79	0.74	0.71	0.57	0.78	0.95	0.77	0.40								
Val	0.81	0.74	0.83	0.91	0.81	0.82	0.82	0.88	0.87	0.70	0.70	0.84							
Phe	0.63	0.59	0.79	0.78	0.72	0.68	0.61	0.76	0.91	0.76	0.41	0.98	0.89						
Ile	0.96	0.93	0.95	0.96	0.96	0.95	0.93	0.98	0.87	0.77	0.89	0.75	0.91	0.77					
Leu	0.78	0.80	0.90	0.95	0.85	0.90	0.78	0.94	0.98	0.68	0.68	0.91	0.89	0.87	0.89				
Orn	0.55	0.51	0.57	0.23	0.57	0.23	0.26	0.31	0.37	0.86	0.32	0.40	0.34	0.43	0.43	0.22			
Lys	0.98	0.95	0.93	0.85	0.97	0.88	0.90	0.91	0.74	0.76	0.93	0.56	0.73	0.55	0.93	0.75	0.52		
Trp	0.79	0.83	0.91	0.87	0.86	0.83	0.65	0.86	0.93	0.85	0.63	0.91	0.88	0.89	0.88	0.91	0.49	0.72	
Non-cleaved	-0.71	-0.60	-0.65	-0.82	-0.67	-0.77	-0.92	-0.81	-0.67	-0.29	-0.76	-0.51	-0.75	-0.54	-0.77	-0.75	0.08	-0.71	-0.49

Supplementary Fig. 5. ROC curve analysis to estimate the presence of (a) high 2-PN rate > 80%; (b) low degeneration rate < 15%; (c) high cleavage rate > 80%; (d); high blastocyst rate > 30%; and (e) incidence of the cleavage. The best cut-off was obtained by maximizing the Youden index.

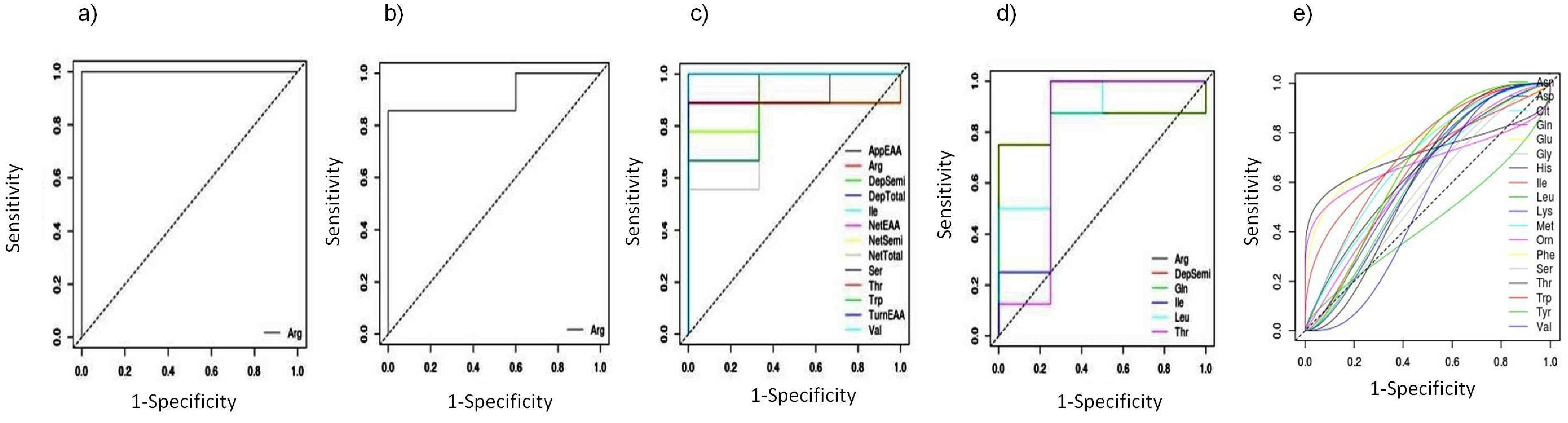

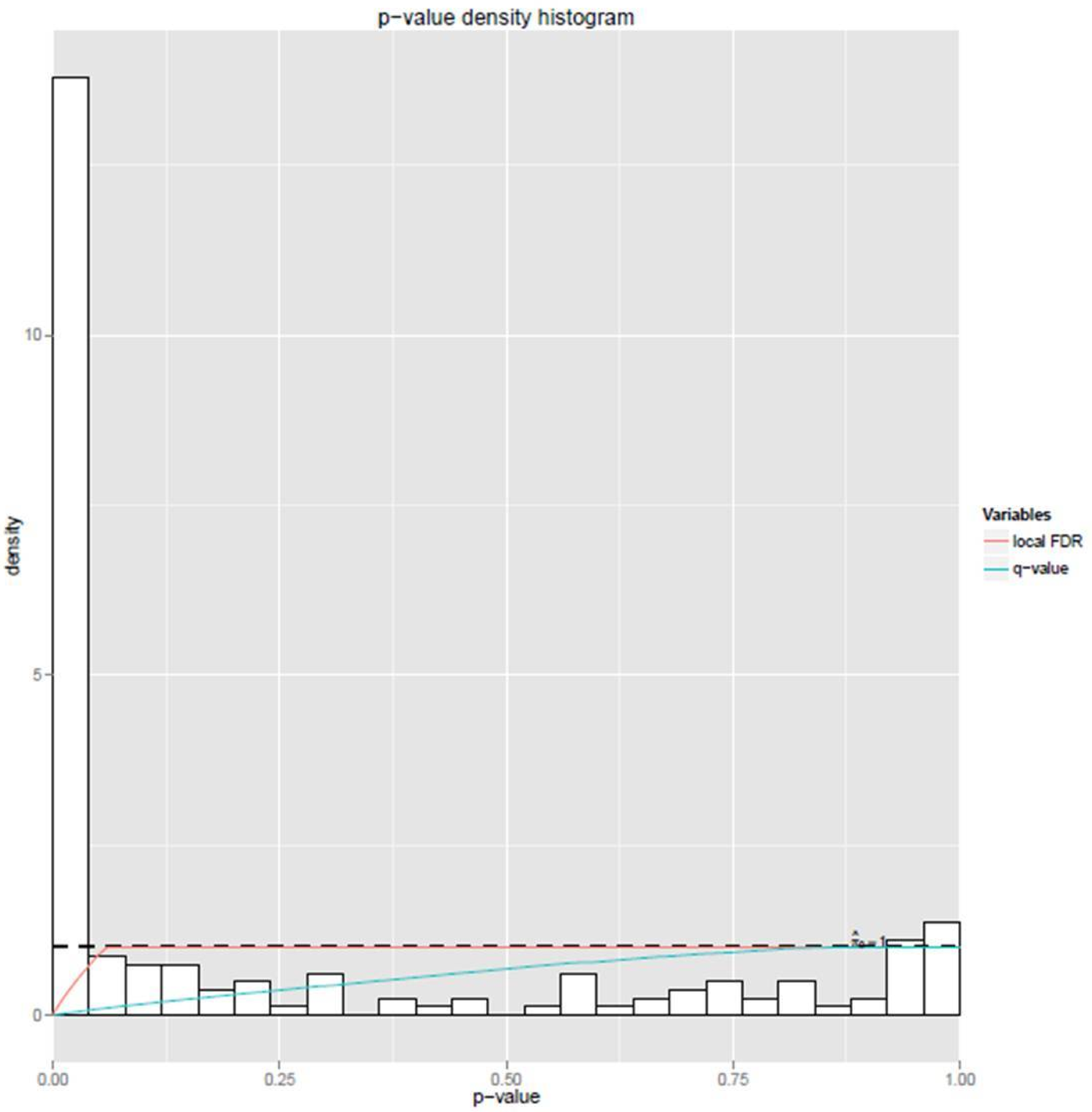

Supplementary Fig. 6. A density histogram of the 220 p values with line plots of the q-values and local False Discovery Rate (FDR) values versus p-values. The π0 estimate is also displayed.

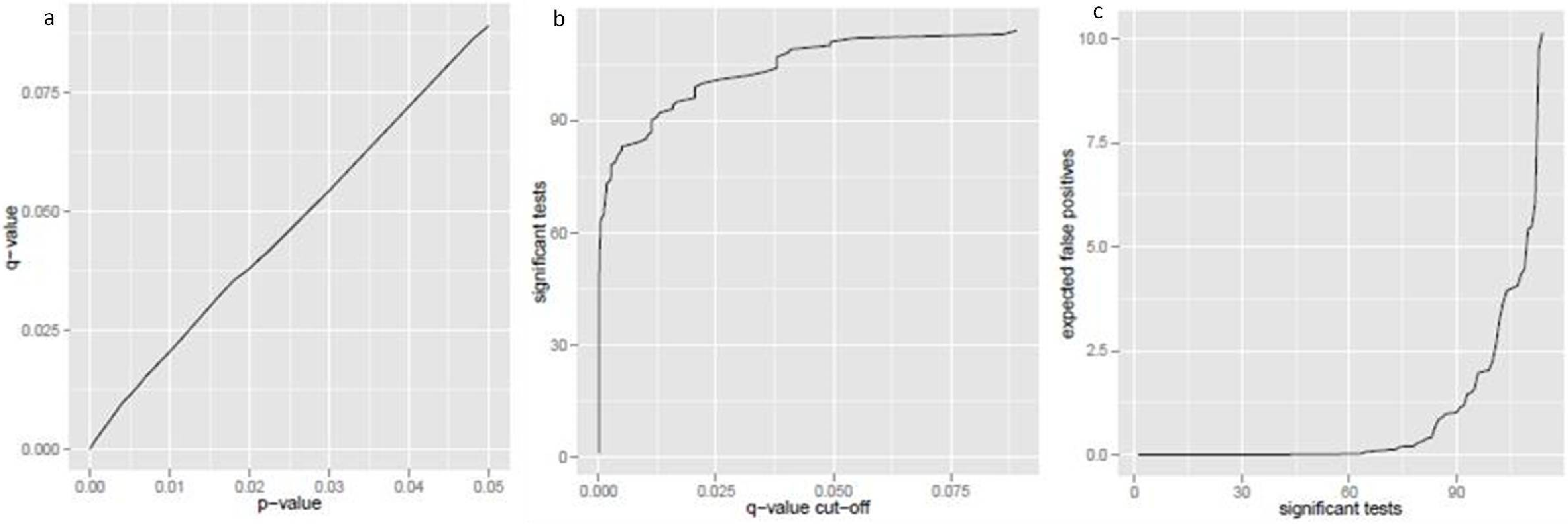

Supplementary Fig. 7. (a) The q-values versus their respective p-values. (**b**) The number of comparisons occurring on the list up through each q-value versus the respective q-value. (**c**) The expected number of false positive comparisons versus the total number of significant comparisons given by the q-values.

Supplementary Table 1. Linear and quadratic effects of the presence of urea during IVM on amino acid depletion/appearance by COCs and subsequent oocyte developmental competence.

	Treatments					Contrast P value		
Item	**Control**	**EGF**	**EGF+U20**	**EGF+U40**	**SEM**	**U^+ vs. U^-**	**Linear**	**Quadratic**
Asp	0.72	0.40	0.37	0.40	0.14	0.94	0.99	0.91
Glu	4.68	2.63	4.54	2.0	0.36	0.43	0.50	**0.02**
Ser	-2.96	-1.46	-2.58	-1.78	0.20	0.16	0.55	0.07
Gln	-7.65	-7.24	-7.69	-9.1	0.27	0.25	0.12	0.62
His	0.07	0.29	0.79	0.08	0.05	0.43	0.33	**0.01**
Gly	7.34	4.43	6.17	2.47	0.37	0.92	0.17	**0.04**
Thr	0.89	1.39	1.30	0.76	0.13	0.24	0.09	0.45
Cit	0.26	0.20	0.30	0.20	0.01	0.05	0.95	**0.02**
Arg	-0.17	0.19	-0.69	-1.19	0.14	0.001	**0.01**	**0.005**
Ala	7.74	6.37	8.97	4.46	0.52	0.74	0.14	**0.01**
Tyr	0.93	0.76	1.23	0.49	0.11	0.67	0.37	**0.03**
Val	0.43	2.38	1.04	0.53	0.20	0.01	**0.006**	0.37
Met	0.14	-0.59	0.15	-0.08	0.09	0.08	0.19	0.16
Trp	0.03	5.05	0.20	0.02	0.53	0.02	**0.03**	0.22
Phe	0.23	0.62	0.70	0.26	0.09	0.47	0.12	0.19
Ile	0.03	0.62	0.45	0.05	0.17	0.19	0.09	0.68
Orn	0.90	0.78	2.14	0.66	0.14	0.009	0.59	**<0.0001**
Leu	0.64	1.01	0.97	0.04	0.25	0.22	0.06	0.29
Lys	1.16	1.02	1.47	-0.10	0.27	0.49	0.12	**0.05**
Total depletion of all AAs	-11.17	-8.70	-11.75	-11.87	0.71	0.04	0.06	0.28
Total appearance of all AAs	16.44	24.73	21.74	12.46	1.74	0.03	**0.001**	**0.006**
Total net balance of all AAs	4.27	12.74	11.99	-1.59	2.17	0.001	**<0.0001**	**0.008**
Total turnover of all AAs	29.62	32.58	31.89	22.32	1.51	0.40	**0.05**	**0.05**
Total depletion of non-EAAs	-0.21	-0.12	-0.34	-0.89	0.10	0.01	**0.009**	0.74
Total appearance of non-EAAs	7.31	5.38	8.97	3.71	0.85	0.49	0.29	**0.009**
Total net balance of non-EAAs	6.31	4.78	6.97	2.71	0.82	0.49	0.29	**0.009**
Total turnover of non-EAAs	7.31	5.38	8.97	4.71	0.88	0.49	0.29	**0.009**
Total depletion of semi-EAAs	-11.83	-8.69	-9.46	-11.57	0.51	0.10	0.14	0.41
Total appearance of semi-EAAs	7.98	5.37	7.40	2.96	0.54	0.76	**0.01**	**0.0009**
Total net balance of semi-EAAs	-3.84	-3.32	-4.05	-8.62	0.85	0.02	**0.002**	0.10
Total turnover of semi-EAAs	21.81	14.07	17.86	14.53	0.62	0.24	0.85	**0.05**
Total depletion of EAAs	-0.40	-0.52	0.0	-0.23	0.44	0.23	0.44	0.26
Total appearance of EAAs	2.24	12.91	7.05	1.78	0.52	0.003	**0.001**	0.89
Total net balance of EAAs	1.84	12.39	7.05	1.54	0.25	0.009	**0.004**	0.97
Total turnover of EAAs	2.64	13.42	7.05	2.01	0.34	0.001	**0.0006**	0.72
2-PN	87.85	93.11	74.02	69.80	1.80	<0.0001	**<0.0001**	0.21
Cleavage rate	68.70	89.42	61.93	55.70	2.28	<0.0001	**<0.0001**	0.08
Blastocyst rate	28.47	31.03	23.48	23.77	1.02	0.05	0.08	0.25
Degeneration rate	12.31	10.14	38.51	44.87	3.01	0.0002	**<0.0001**	0.35

Least squares means and SEM are presented in the tables. U^+: presence of urea; U^-: absence of urea; U20: 20 mg/dl urea; U40: 40 mg/dl urea. Significance was declared at $P \leq 0.05$.

Supplementary Table 2. The principal component analysis (PCA) was performed in order to reduce the broad set of variables (high dimensional data) and to consider a concurrent association between AAs and oocyte competence.

	COCs Individually Non-cleaved			COCs Individually cleaved			COCs matured In groups		
	PC 1	PC 2	PC 3	PC 1	PC 2	PC 3	PC 1	PC 2	PC 3
Asp	0.24	-0.05	0.24	0.07	0.11	0.51	0.10	-0.11	-0.47
Glu	0.23	-0.02	0.23	0.13	0.39	-0.09	0.26	-0.20	-0.20
His	0.25	0.09	0.08	0.28	0.07	-0.18	-0.10	0.27	0.33
Ser	0.24	-0.13	-0.12	0.27	-0.23	0.01	0.11	0.19	0.09
Gln	0.24	0.03	0.16	0.30	0.08	-0.02	0.02	-0.11	0.30
Arg	0.24	-0.17	-0.02	0.22	-0.28	0.21	0.27	-0.09	-0.22
Cit	0.22	-0.27	0.08	0.19	-0.09	-0.29	0.29	0.09	0.13
Gly	0.25	-0.11	-0.04	0.30	-0.11	0.10	0.22	-0.26	0.07
Thr	0.23	0.11	-0.23	0.25	0.01	0.34	0.07	0.35	-0.19
Ala	0.20	0.40	0.17	0.09	0.42	0.04	0.31	-0.12	0.02
Tyr	0.21	-0.26	0.29	0.12	0.10	0.45	0.29	-0.15	0.03
Met	0.21	0.26	-0.36	0.27	0.07	-0.27	0.09	0.28	0.31
Val	0.23	0.01	-0.20	0.29	-0.12	-0.07	0.09	-0.18	0.04
Phe	0.20	0.25	-0.35	0.28	0.15	-0.17	0.05	0.32	0.17
Ile	0.25	-0.06	0.05	0.30	-0.03	-0.15	0.30	0.03	0.15
Leu	0.23	-0.02	-0.27	0.28	-0.19	-0.08	0.27	0.11	0.15
Orn	0.12	0.51	0.42	0.06	0.42	-0.05	0.23	-0.22	0.26
Lys	0.23	-0.09	0.29	0.17	-0.20	0.27	0.31	0.06	0.04
Trp	0.23	0.21	-0.14	0.07	0.41	0.13	0.32	0.00	0.08
Non-cleaved	-0.19	0.41	0.14						
Cleaved				0.18	0.16	0.14			
2PN rate							0.15	0.29	-0.23
Cleavage rate							0.13	0.33	-0.06
Blastocyst rate							0.15	0.21	-0.18
Degeneneration rate							-0.13	-0.27	0.29
Eigenvalue	15.94	1.83	1.5	9.71	5.03	3.39	9.2	6.03	2.67
Variance %	79.7	9.2	7.5	48.5	25.1	17	40	26.2	11.6
Accumulative variance %	79.7	88.9	96.4	48.5	73.6	90.6	40	66.2	77.8
